# Oxidative Stress Resistance in Metastatic Prostate Cancer: Renewal by Self-Eating

**DOI:** 10.1371/journal.pone.0145016

**Published:** 2015-12-15

**Authors:** Jan Balvan, Jaromir Gumulec, Martina Raudenska, Aneta Krizova, Petr Stepka, Petr Babula, Rene Kizek, Vojtech Adam, Michal Masarik

**Affiliations:** 1 Department of Pathological Physiology, Faculty of Medicine, Masaryk University / Kamenice 5, CZ-625 00, Brno, Czech Republic; 2 Central European Institute of Technology, Brno University of Technology, Technicka 3058/10, CZ-616 00, Brno, Czech Republic; 3 TESCAN Brno, s.r.o., Brno, Czech Republic; 4 Department of Physiology, Faculty of Medicine, Masaryk University / Kamenice 5, CZ-625 00, Brno, Czech Republic; 5 Department of Chemistry and Biochemistry, Mendel University in Brno / Zemedelska 1, CZ-613 00, Brno, Czech Republic; University of Kentucky College of Medicine, UNITED STATES

## Abstract

Resistant cancer phenotype is a key obstacle in the successful therapy of prostate cancer. The primary aim of our study was to explore resistance mechanisms in the advanced type of prostate cancer cells (PC-3) and to clarify the role of autophagy in these processes. We performed time-lapse experiment (48 hours) with ROS generating plumbagin by using multimodal holographic microscope. Furthermore, we also performed the flow-cytometric analysis and the qRT-PCR gene expression analysis at 12 selected time points. TEM and confocal microscopy were used to verify the results. We found out that autophagy (namely mitophagy) is an important resistance mechanism. The major ROS producing mitochondria were coated by an autophagic membrane derived from endoplasmic reticulum and degraded. According to our results, increasing ROS resistance may be also accompanied by increased average cell size and polyploidization, which seems to be key resistance mechanism when connected with an escape from senescence. Many different types of cell-cell interactions were recorded including entosis, vesicular transfer, eating of dead or dying cells, and engulfment and cannibalism of living cells. Entosis was disclosed as a possible mechanism of polyploidization and enabled the long-term survival of cancer cells. Significantly reduced cell motility was found after the plumbagin treatment. We also found an extensive induction of pluripotency genes expression (*NANOG*, *SOX2*, and *POU5F1*) at the time-point of 20 hours. We suppose, that overexpression of pluripotency genes in the portion of prostate tumour cell population exposed to ROS leads to higher developmental plasticity and capability to faster respond to changes in the extracellular environment that could ultimately lead to an alteration of cell fate.

## Introduction

Prostate cancer (PC) is one of the most frequently diagnosed cancer types in men. Most of prostate cancers are initially responsive to androgen deprivation therapy, but later would emerge an aggressive, androgen-independent phenotype resistant to conventional therapies. This advanced type of prostate cancer easily metastasizes. Hematogeneous metastases are usually present in 35% of prostate cancer patients with the most frequent localisation in bones (90%). The widely studied model for androgen-independent, advanced, metastases-producing prostate cancer is the PC-3 cell line established from the lumbar metastasis of a 62 year old Caucasian male with grade 4 of prostatic adenocarcinoma. PC-3 cells are hemizygous for 17p chromosome, and their sole copy of the *p53* gene has a stop codon at position 169 [1]. As a result, PC-3 cells do not express the functional p53 protein, which makes it rather resistant to p53-mediated apoptosis [2]. Furthermore, we chose PC-3 cell line and not DU145, because DU145 prostate cancer cells express PTEN, which is not expressed by PC-3 cells [3, 4]. Multiple functional studies support the role of PTEN as a critical tumour suppressor in prostate cancer [5–7].

In our previous study we demonstrated that the PC-3 cell line showed higher resistance to cisplatin-induced apoptosis and no decreasing proportion of G2/M fraction (4N DNA content) evident in 22Rv1 cells [8]. Cisplatin is primarily considered as a DNA-damaging agent, forming different types of hard-reparable adducts with cellular DNA [9]. Apart from DNA damage, cisplatin also induces reactive oxygen species (ROS) [10]. Due to the fact, we have focused on another ROS-producing reagent, plumbagin [11], which does not form DNA adducts, to assess importance of cell death modulation and dealing with ROS for PC-3 resistance. Plumbagin (5-hydroxy-2-methyl-1,4-naphthoquinone) occurs naturally in the medicinal herb *Plumbago zeylanica L*. and belongs to naphthoquinones. Naphthoquinones display their cytotoxic actions through two ways: as pro-oxidants, reducing oxygen to reactive oxygen species; and as electrophiles, which form covalent bonds with tissue nucleophiles [12]. Furthermore, plumbagin was also shown to suppress the activation of nuclear factor-κB (NF-κB) [13].

In recent studies, ROS generation was associated with the mitochondria as a consequence of impaired mitochondrial protein synthesis [10]. Furthermore, it was pointed out, that cells with a deficit of functional mitochondria are more resilient to cell damage by cisplatin [14]. However, Panov *et al*. found out, that the prostate cancer cell lines LNCaP, PC-3, and DU145 contained 2 to 4 times more mitochondria per gram of cells than normal prostate epithelial cells. Respiratory activities of mitochondria isolated from normal prostate epithelial cells were also 5-20-fold lower than those of mitochondria isolated from prostate cancer cells [15]. Therefore, we presume the existence of some protective mechanisms against ROS in PC-3 cells. Many cell injuries caused by ROS could be sublethal (especially if the studied cells have disrupted apoptosis-triggering mechanisms) and result in an altered steady state in which the damaged cells are able to survive. Even if a damaged cell is driven to oncosis (oncosis is a pre-lethal phase that follows a serious cell injury) or senescence, there are probably some mechanisms to reverse this process [16–18], particularly if the cell is able to get rid of damaging factors and restore ATP production. A possible way to gain enough energy for the survival could be autophagy [19], cannibalism or entosis [20, 21]. Autophagy was at first considered a mechanism that suppresses malignant transformation. However, strong evidences for a dual role of autophagy were discovered [22]. In early tumours, autophagy could be truly a potent tumour suppressor because it can assure organelle and protein quality control and prevent genomic instability and aneuploidy organelle [23]. However, there are significant evidences that autophagy has a cancer-promoting role in established tumours [24].

One of the significant histopathological features of human solid tumours is the occurrence of large atypical cancer cells with multiplicated nuclear DNA that are known as polyploid giant cancer cells (PGCCs). Increased PGCCs numbers usually appear in late disease stages and grades or as a consequence of chemotherapy [25]. An important goal of our study was to explore possible mechanisms of defence against ROS in the advanced type of prostate cancer cells. We tried to assess the role of autophagy and the formation of polyploid giant cancer cells (PGCCs) in the advanced type of prostate cancer. We also attempted to verify a hypothesis that autophagy could be the mechanism of resistance against ROS rather than the mechanism of cell death. This hypothesis is supported by recent findings indicating that well-characterized autophagy activators and mTOR inhibitors (such as rapamycin, PP242, or resveratrol) markedly improve the speed and efficiency of puripotent stem cells generation [26].

## Materials and Methods

### Chemical and biochemical reagents

Ham’s F12 medium, fetal bovine serum (FBS), (mycoplasma free), penicillin/streptomycin and trypsin were purchased from PAA Laboratories GmbH (Pasching, Austria). Phosphate-buffered saline (PBS) was purchased from Invitrogen Corp. (Carlsbad, CA, USA). Ethylenediaminetetraacetic acid (EDTA), plumbagin and other chemicals of ACS purity were purchased from Sigma-Aldrich Co. (St. Louis, MO, USA), unless noted otherwise.

### Cell cultures and cultured cell conditions

Human PC-3 prostate cancer cells were used in this study (passage 18–24). The PC-3 cell line was established from grade 4 prostatic adenocarcinoma from 62 years old Caucasian male and derived from the metastatic site in bones. The PC-3 cell line was purchased from HPA Culture Collections (Salisbury, UK).

PC-3 cells were cultured in Ham’s F12 medium with 7% FBS. The medium was supplemented with penicilin (100 U/ml) and the cells were maintained at 37°C in humidified incubator with 5% CO_2_. Hypoxy/starvation-resistant PC-3 were selected by cultivation without the access of oxygen and with no medium replacement for one month.

### Plumbagin treatment

The stock solution of plumbagin was prepared in dimethylsulfoxide (DMSO) and diluted with the medium. An equal volume of DMSO (final concentration ≤ 0.1%) was added to the controls. The plumbagin treatment was initialized after the cells reached confluence of ~50%. For cytotoxicity assessment, a range of concentrations 0, 0.5, 1, 1.5, 2, 2.5, 3, 4, 5, and 6 μmol/l of plumbagin was used. Time points for cell harvesting and thus for subsequent analyses were 0 h, 40 min., 1,5, 4, 6, 8, 10, 16, 20, 24, 36, and 48 h.

### Cell content quantification

Total cell content was measured using Casy model TT system (Roche Applied Science, USA) and the following protocol: first, calibration was performed from the samples of viable and necrotic cells. For the necrotic cells, 100 μl cell suspension and 800 μl Casy Blue solution were mixed and left for 5 minutes at room temperature. Subsequently, 9 ml CasyTone was added. To prepare a viable cell standard, 100 μl of cell suspension was mixed with 10 ml CasyTone. All subsequent measurements were performed on 100x diluted 100 μl cell suspension. Prior to each measurement, the background was subtracted. All samples were measured in duplicates.

### Measurement of cell viability—MTT test

The suspension of 5000 cells was added to each well of standard microtiter plates. Volume of 200 μl was transferred to wells 2–11. The medium (200 μl) was added to the first and to the last column (1 and 12, control). The plates were incubated for 2 days at 37°C to ensure cell growth. The medium was removed from columns 2 to 11. Columns 3–10 were filled with 200 μl of medium containing an increased concentration of plumbagin (0–6 μmol/l). As a control, columns 2 and 11 were filled with the medium without plumbagin. The plates were incubated for 12 and 24 h, then the medium was removed and the cells were washed in PBS. Columns 1–11 were filled with 200 μl of medium containing 50 μl of MTT (5mg/ml in PBS), incubated in humidified atmosphere for 4 h at 37°C, and wrapped in aluminium foil. After the incubation, the MTT-containing medium was replaced with 200 μl of 99.9% dimethylsulfoxide (DMSO) to dissolve MTT-formazan crystals. Subsequently, 25 μl of glycine buffer was added to all wells and absorbance was determined immediately at 570 nm (VersaMax microplate reader, Molecular Devices, Sunnyvale, CA, USA).

### Cell growth and proliferation assay using impedance measurement

Cell growth was analyzed using the real-time cell analysis (RTCA) system (xCELLigence; Roche Applied Science and ACEA Biosciences). Firstly, the optimal seeding concentration for proliferation and cytotoxic assay was determined. PC-3 cells were seeded at a density of 7000 cells per well in E-Plates 16. After seeding the total number of cells in 200 μl medium to each well, in E-Plate 16, the attachment, proliferation and spreading of the cells was monitored every 15 min. After 24 hours, plumbagin was added and the cell index (CI), which reflects cell viability, was monitored. All experiments were carried out for 250h. The results are expressed as a cell index using the manufacturer’s software (Roche Applied Science and ACEA Biosciences). The experiments were made in duplicates.

### Flow cytometric analysis of cell death

Double-staining with fluorescein isothiocyanate (FITC)/propidium iodide (PI) was undertaken using the Annexin V-FLUOS-staining kit (Roche Applied Science) according to the manufacturer’s protocol in order to determine percentages of viable, apoptotic and necrotic cells following the exposure to plumbagin. Briefly, the cells were harvested by repetitive pipetting and washed two times with PBS (centrifuged at 2000 rpm for 5 min), resuspended in 100 μl of Annexin-V-FLUOS labelling solution and incubated for 15 min. in the dark at 15–25°C. Annexin V-FITC binding was detected by flow cytometry (Partec GmbH, Münster, Germany) (Ex = 488 nm, Em = 533 nm, FL1 filter for Annexin-V-FLUOS and FL3 filter for PI).

### Flow cytometric detection of autophagosomes

Autophagosome formation in PC-3 cells was detected using the CYTO-ID Autophagy Detection Kit (Enzo, PA, USA) following the manufacturer’s instruction. The CYTO-ID green fluorescent reagents specifically detect acid autophagic vacuoles formed during autophagy. Briefly, the cells were harvested by gentle repetitive pipetting, spun down and washed twice in RPMI 1640 with 5% fetal bovine serum (FBS). The cells were resuspended in 500 μl of freshly diluted CYTO-ID staining reagent and incubated in the dark at 37°C for 30 min. CYTO-ID fluorescence of cells was immediately analyzed by flow cytometry using the flow cytometr (Partec GmbH, Münster, Germany) (Ex = 480 nm, Em = 530 nm, FL1 filter for CYTO-ID, SSC for cellular granularity). The percentage of cells with CYTO-ID staining was used to represent the formation of autophagosomes.

### Flow cytometric analysis of intact healthy cells

The cell pellet was prepared as mentioned above. The cells were resuspended in 500 μl of freshly diluted 1 μM SYTO 16 (Thermo Fisher Scientific, Waltham, MA, USA) staining solution and incubated in the dark at 37°C for 45 min. SYTO 16 fluorescence of cells was immediately analyzed on the same instrument as mentioned above (Ex = 488 nm, Em = 518 nm, FL1 filter for SYTO 16, FSC for cell size). The percentage of SYTO 16-positive cells was analyzed. The data were analyzed using the FloMax software (Partec GmbH, Münster, Germany).

### Fluorescence microscopy and cell staining

For fluorescence microscopy, the cells were cultivated directly on microscope glass slides (75x25 mm, thickness 1mm, Menzel Glässer, Braunschweig, Germany) in Petri dishes in the above-described cultivation media (see Cultured cell conditions). The cells were transferred directly onto the slides, which were submerged in the cultivation media. After the treatment, the microscope glass slides with a monolayer of cells were removed from the Petri dishes, rinsed in the cultivation medium without plumbagin supplementation and PBS buffer and directly used for staining and fluorescence microscopy.

The cells were incubated with the following highly specific fluorescent probes: reactive oxygen species were visualized using CellROX Deep Red reagent (Life Technologies, USA, 5 μM, cell-permeant, life-cell stain with absorption/emission maxima of 644/665 nm), mitochondria were visualized using MitoTracker Green FM (Life Technologies, USA, 300 nM, cell-permeant life-cell stain with absorption/emission maxima of 490/516 nm), and endoplasmic reticulum was visualized using ER-Tracker Red (Life Technologies, USA, 1 μM, cell-permeant, life-cell stain with absorption/emission maxima of 587/615 nm). After incubation (45 min, 37°C, dark), the cells were washed three times with PBS buffer (0.05 M, pH 7.0) and observed under the confocal microscope (Leica TCS SP8 X, Germany) using appropriate excitation and emission wavelengths.

### TEM visualization of PC-3 ultrastructure

The PC-3 cells were gently harvested by repetitive pipetting and spun down (2000 rpm, 5 min.). Briefly, the cells were fixed with 3% glutaraldehyde in cacodylate buffer for 2 hours and washed three times for 30 minutes in 0.1 M cacodylate buffer. Following this, they were fixed with 0.02 M OsO_4_ dissolved in 0.1 M cacodylate buffer, dehydrated in alcohol, and infiltrated with acetone and No. 1 Durcuptan mixture overnight. On the following day, the cells were infiltrated with No. 2 Durcuptan mixture, embedded and polymerized. Ultrathin sections (90 nm, Ultramicrotome LKB, Bromma, Stockholm, Sweden) were transferred onto grids covered with the Formvar membrane (Marivac Ltd., Halifax, Canada). 2% uranyl acetate and Reynold’s solution were used for contrast staining. The sections were viewed in the transmission electron microscope (Morgagni 268, FEI Europe B.V., Eindhoven Netherlands). Software AnalySIS (Soft Imaging System, GmbH, Münster, Germany) was used for image analysis of cell ultrastructure.

### Quantitative phase imaging

Quantitative phase imaging is a non-invasive technique with high intrinsic contrast even for naturally transparent objects such as live cells. Therefore this method is suitable for long term observations of cell reactions to treatment without any additional staining. In these experiments quantitative phase imaging was performed by Tescan multimodal holographic microscope Q-PHASE. Q-PHASE is based on the original concept of coherence-controlled holographic microscope [27, 28].

Quantitative phase imaging was initiated immediately after plumbagin treatment. Cells were cultivated in Flow chambers μ-Slide I Lauer Family (Ibidi, Martinsried, Germany) In order to image enough number of cells in one field of view, objectives Nikon Plan 10/0.30 were chosen. Holograms were captured by CCD camera (XIMEA MR4021 MC-VELETA). The entire image reconstruction and image processing were performed in Q-PHASE control software. Quantitative phase images are shown in grayscale with units of pg/μm^2^ that were recalculated from original radians according to Barer and Davies [29, 30]. Movies with identification arrows were prepared in ImageJ software.

### RNA Isolation and Reverse Transcription

TriPure Isolation Reagent (Roche, Basel, Switzerland) was used for RNA isolation. RNA samples without reverse transcription were used as negative control for qRT-PCR to exclude DNA contamination. The isolated RNA was used for the cDNA synthesis. RNA (1000 ng) was transcribed using the transcriptor first strand cDNA synthesis kit (Roche, Switzerland), which was applied according to manufacturer's instructions. The cDNA (20 μl) prepared from the total-RNA was diluted with RNase-free water to 100 μl and the amount of 5 μl was directly analyzed by using the LightCycler^®^480 II System (Roche, Basel, Switzerland).

### Quantitative real-time polymerase chain reaction

qRT-PCR was performed using TaqMan gene expression assays and the LightCycler^®^480 II System (Roche, Basel, Switzerland). The amplified DNA was analyzed by the comparative Ct method using β-actin as a reference gene. The primer and probe sets for ACTB (assay ID: Hs99999903_m1), BECN1 (Hs00186838_m1), BIRC5 (Hs00153353_m1), CCL2 (Hs00234140_m1), MAP1LC3 (Hs00797944_s1), SOX2 (Hs01053049_s1), NANOG (Hs04260366_g1), POU5F1 (Hs04260367_gH), and HIF1A (Hs00153153_m1) were selected from the TaqMan gene expression assays (Life Technologies, USA). The qRT-PCR was performed under the following amplification conditions: total volume of 20 μl, initial incubation at 50°C/2 min followed by denaturation at 95°C/10 min, then 45 cycles at 95°C/ 15 sec and at 60°C/1 min.

### Statistics

Pearson correlation, principal component analysis and cluster analysis were performed to reveal associations between cases and variables. These analyses were performed on standardized data; the cluster analysis was performed using Ward’s method. All charts are depicted with means and standard deviations. Software Statistica (StatSoft, Tulsa, OK, USA) was used for analysis.

## Results

### Determination of IC50 for plumbagin

**Fig 1:**
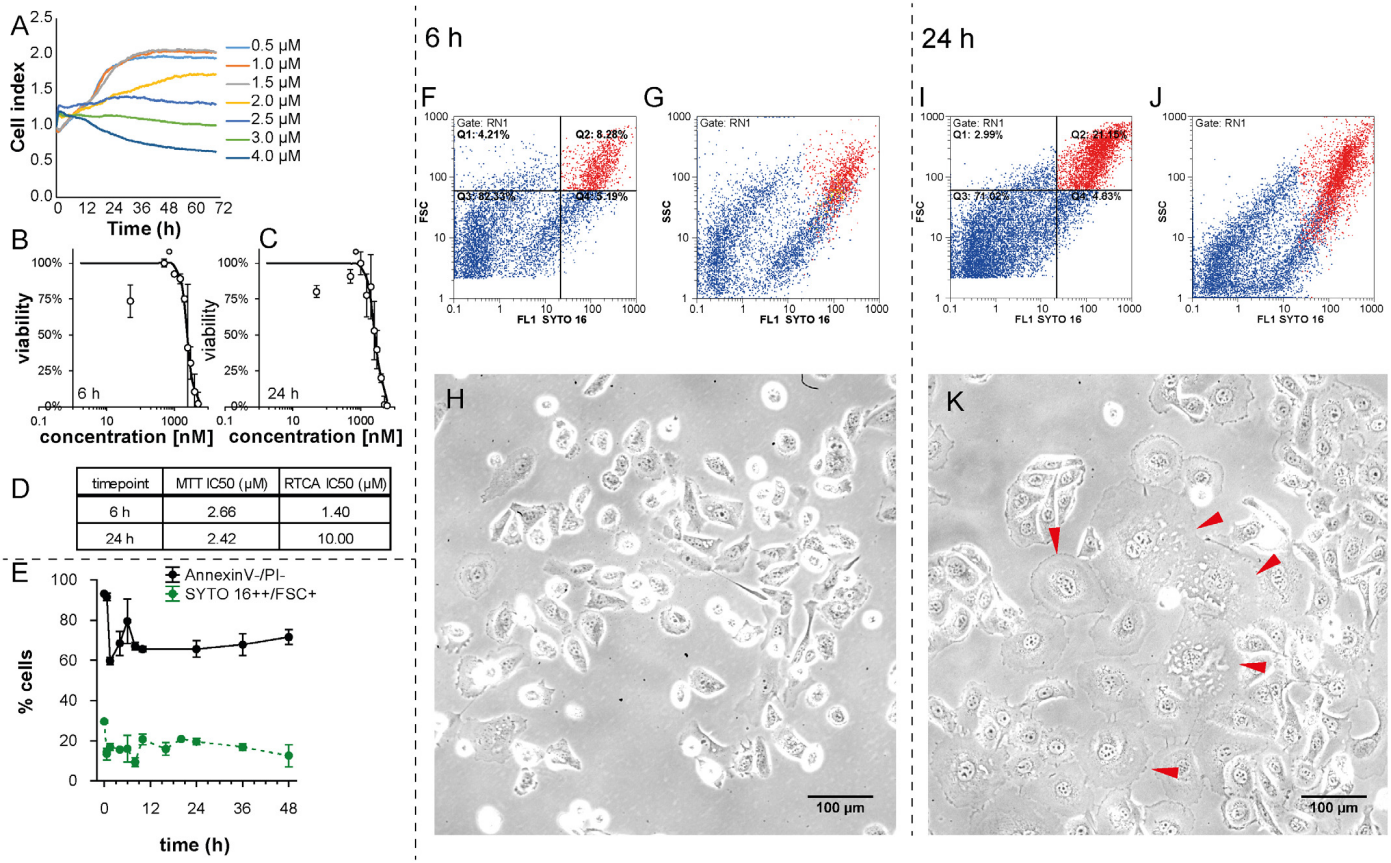
Effect of plumbagin treatment on viability and size of cells. **(A)** Real-time monitoring of relative cell impedance (showed as a cell index) using the RTCA system. **(B)** MTT-assessed response to 6h plumbagin treatment. **(C)** MTT-assessed response to 24h plumbagin treatment. **(D)** IC50 values according to RTCA and MTT and length of treatment. **(E)** Time-dependent changes in the quantity of large cells with intact nuclei (SYTO 16++/FSC+) and intact cells (AnnexinV-/PI-) assessed by flow-cytometry. **(F)** Numbers of large healthy cells depicted as SYTO 16++ /FSC+ (red) cluster at flow-cytometric dot plot at 6h time-point; Forward-scattered light (FSC) is proportional to cell-surface area or size. **(G)** Granularity of large healthy cells depicted as SYTO 16++ /SSC+ (red) cluster at flow-cytometric dot plot at 6h time-point; Side-scattered light (SSC) is proportional to cell granularity or internal complexity. **(H)** Morphology of PC-3 cells after 6h plumbagin treatment, 20x magnification, phase contrast microscopy. **(I)** Numbers of large healthy cells depicted as SYTO 16++/FSC+ (red) cluster at flow-cytometric dot plot at 24h time-point; Forward-scattered light (FSC) is proportional to cell-surface area or size. **(J)** Granularity of large healthy cells depicted as SYTO 16++/SSC+ (red) cluster at flow-cytometric dot plot at 24h time-point; Side-scattered light (SSC) is proportional to cell granularity or internal complexity. **(K)** Morphology of PC-3 cells after 24h plumbagin treatment; giant PC-3 cells with polyploid giant cancer cell (PGCCs)-like morphology are highlighted by arrows. 20x magnification, phase contrast microscopy.

To assess the cytotoxic effect of plumbagin on the PC-3 cell line, and to select concentrations for further analyses, MTT test and real time cell analysis (RTCA) impedance based test were performed with concentrations 0 (no drug added), 0.5, 1, 1.5, 2, 2.5, 3, 4, 5, and 6 μmol/l. Using the logistic regression, IC50 concentrations were determined at time-points 6 and 24h (see Fig 1A, 1B, 1C and 1D). The output of RTCA method is a cell index value (CI), see Fig 1A, that reflects the number of cells, as well as morphological parameters, such as the size, shape, and degree of cell attachment to the substrate. This means that an increase in the average size of surviving cells could affect the CI value and could correlate with higher IC50 values (1.4 μM and 10 μM IC50 after 6h and 24h treatment, respectively). We performed the flow-cytometric analysis by using SYTO 16 double-positivity as a marker of viable cells and forward scatter (FSC) to detect the size of surviving cells after the 2 μM plumbagin treatment. Numbers of large healthy cells depicted as a SYTO 16++ /FSC+ (red) cluster at the flow-cytometric dot plot were elevated at 24h time-point in comparison with 6h time-point; see Fig 1F, 1G, 1I and 1J. This experiment was done in triplicates. The average rate of large SYTO 16++ was 15.92% after 6h of plumbagin treatment and 19.58% after 24h of plumbagin treatment (Fig 1E). As we observed no dividing cells during the treatment (compare treated and untreated time-lapse, S1 and S5 Videos; cell division was apparent only in untreated cells), emergence of larger cancer cells could be assumed namely because no increase in the percentage of annexin V-/propidium iodide (PI)- cells (healthy cells that would result from dividing) was observed between 5h time-point and 24h time-point of the treatment (Fig 1E). According to these results, the shift in IC50 values identified by the RTCA method may reflect the increased cell size between 6h and 24h time-points, as corroborated by the microscopic analysis; see Fig 1H and 1K.

### PC3 cell line is predisposed to mitophagy

To assess the relative intensity of mitophagy related genes expression (*PINK1*, *FUNDC1*, *SMURF1*, and *PARL*) in the PC-3 cell line, we used the CellMiner Database (http://discover.nci.nih.gov/cellminer/). It allows to precisely determine selected genes expression patterns from 5 microarray platforms in 60 cell lines (NCI60 panel) (S1 Appendix). Pro-mitophagic genes *PINK1*, *FUNDC1*, and *SMURF1* were relatively overexpressed in PC-3 as compared with other cell lines; on the other hand, *PARL* (responsible for PINK1 cleavage) was underexpressed. These data suggest that PC-3 cells have possibly a high level of mitochondrial quality control and are able to effectively identify and then degrade damaged mitochondria.

### Endoplasmic reticulum-affected mitophagy

**Fig 2:**
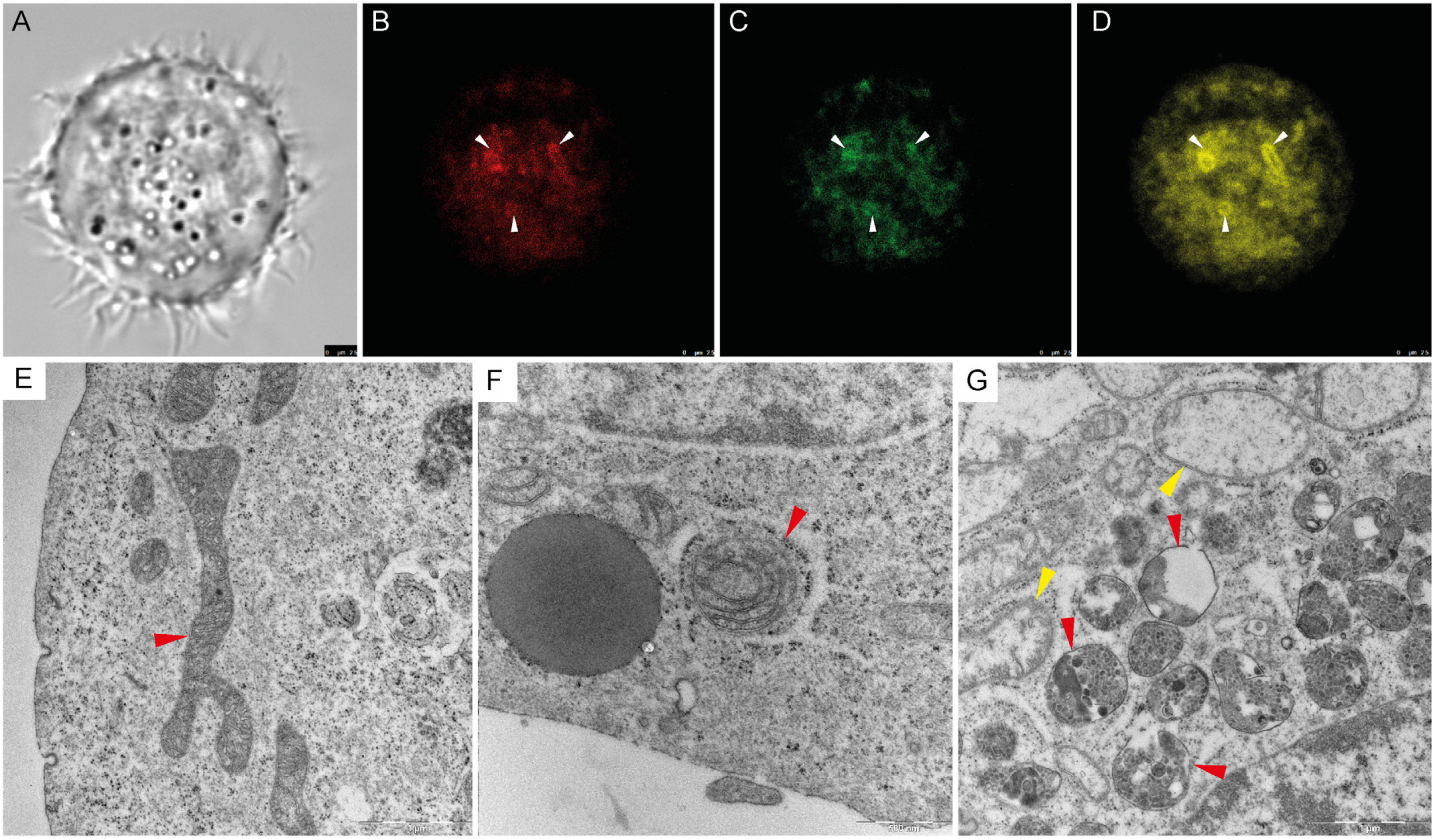
Reactive oxygen species (ROS)-induced mitophagy. **(A)** Phase contrast microscopy of PC-3 cell after plumbagin treatment. **(B)** General accumulation of ROS after plumbagin treatment monitored by confocal microscopy by using CellROX Deep Red Reagent. Areas with ROS accumulation are highlighted by arrows. **(C)** Mitochondria staining monitored by confocal microscopy using MitoTracker Green; area associated with ROS in Fig 2B are highlighted by arrows. **(D)** Endoplasmic reticulum (ER) staining monitored by confocal microscopy using ERTracker Red; areas associated with ROS in Fig 2B are highlighted by arrows. **(E)** Untreated PC-3 cell, cross-section of undamaged mitochondria (highlighted by red arrow); Transmission Electron Microscope (TEM) visualization. **(F)** plumbagin-treated PC-3 cell, mitochondria coated by ER membrane with ribosomes (highlighted by red arrow); TEM visualization. **(G)** Plumbagin-treated PC-3 cell, gradual degradation of mitochondria in autophagosomes visualised by TEM (red arrows); Swollen mitochondria as a marker of damage (yellow arrow).

In order to establish whether the majority of reactive oxygen species (ROS) in the cell is produced by the mitochondria, we applied fluorescent staining after the plumbagin treatment. General accumulation of ROS was monitored using CellROX Deep Red Reagent. Clear colocalisation of ROS and mitochondria staining was found (see Fig 2B and 2C). Major ROS producing mitochondria (see arrows) were coated by isolation membrane derived from ER (see Fig 2D). This observation was corroborated by transmission electron microscopy (TEM) (see Fig 2F). Swollen and damaged mitochondria were wrapped by engulfing membrane and gradually degraded (see Fig 2G). No coating membrane was found around the healthy mitochondria (see Fig 2E).

### Time-lapse imaging

**Fig 3:**
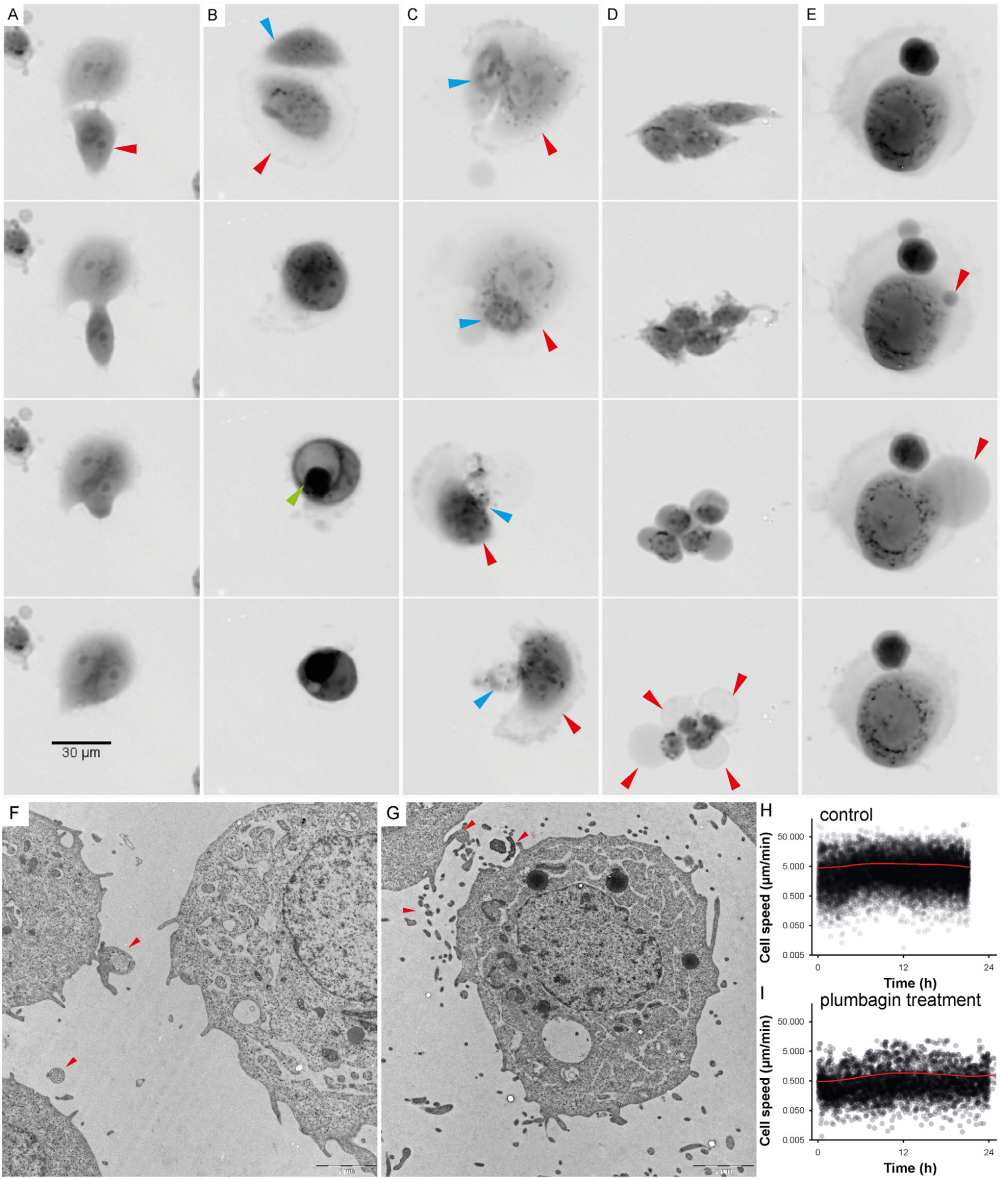
Time-lapse of cell interactions. For detailed time-lapse Videos see S1–S4 Videos. **(A)** Time-lapse imaging of entosis; internalized cell (red arrow) played an active role in its engulfment, which resulted in complete internalization. Both types of cells (engulfing and engulfed) were viable for a long time and lived by about five hours longer than the other observed plumbagin-treated tumour cells. **(B)** Time-lapse imaging of cell fusion with cannibalism (digestion of engulfed cell); during fusion-cannibalism of living cells, the cannibalic cell (red arrow) came in contact with the target cell (blue arrow). The next step was gradual engulfment of the target cell. The nucleus of the target cell appeared initially unaltered whereas the engulfing cell’s nucleus began to change into a semilunar shape. Bird eye structure was observed as a consequence of target cell vacualisation (see green arrow). **(C)** Time-lapse imaging of cannibalism without fusion; the dying cell (blue arrow) was attacked and exploited by the cannibalic cell (red arrow). The target cell was dead after the attack. **(D)** Time-lapse imaging of oncosis; oncotic cells formed typical cytoplasmic blebs that usually lead to necrosis (see red arrow). **(E)** Time-lapse imaging of reverse oncosis; initial forming of oncotic blebs (see red arrow) did not lead to necrosis; the bleb was absorbed and the cell remained viable. (**A-E**) Multimodal holographic microscopy, 10x magnification. **(F)** Communication between PC-3 cells; visualised by TEM (see red arrows). **(G)** Vesicular transfer between PC-3 cells; visualised by TEM (see red arrows). **(H)** Speed of the migration of untreated PC-3 cell population; assessed from holographic microscopy data by CellProfiller software by measurement of “distance travelled”parameter. **(I)** Speed of the migration of PC-3 cell population after 2 μM plumbagin treatment.

A time-lapse Video was captured by holographic microscope to observe the intensity of cell migration and also to quantify the kinetics of PC-3 cells death in 48 hour period. Many different types of cell-cell interactions were monitored and identified during this period including vesicular transfer (Fig 3F and 3G), eating of dead or dying cells (frequency of observation 2.5%; Fig 3C, S3 Video) and engulfment and cannibalism of living cells (frequency of observation 0.8%; Fig 3B). During the cannibalism of living cell, a cannibalic cell came into contact with a target cell. The next step was a gradual engulfment of target cell. The nucleus of the target cell appeared initially unaltered whereas the engulfing cell’s nucleus began to change into a more semilunar shape. Bird eye structure typical for cannibalism was observed (Fig 3B, S2 Video). Finally, the target cell died off. The 2 μM plumbagin treatment had a particular impact on cell motility and on changes in cell-to-cell communication. A significant reduction of cell motility and communication was found after the plumbagin treatment (see Fig 3H and 3I, S1 and S5 Videos).

In oncosis, early changes included marked alterations in the cell shape and volume (Fig 3D, S1 Video). Oncotic cells formed cytoplasmic blebs and showed chromatin clumping followed by necrotic features such as cells membrane rupture and detachment from the surface. Nevertheless, some oncotic cells escaped this fate and were able to reverse processes leading to necrosis. Triggering of oncosis is not an irreversible process; oncosis can be reverted (see Fig 3E, S4 Video).

**Fig 4:**
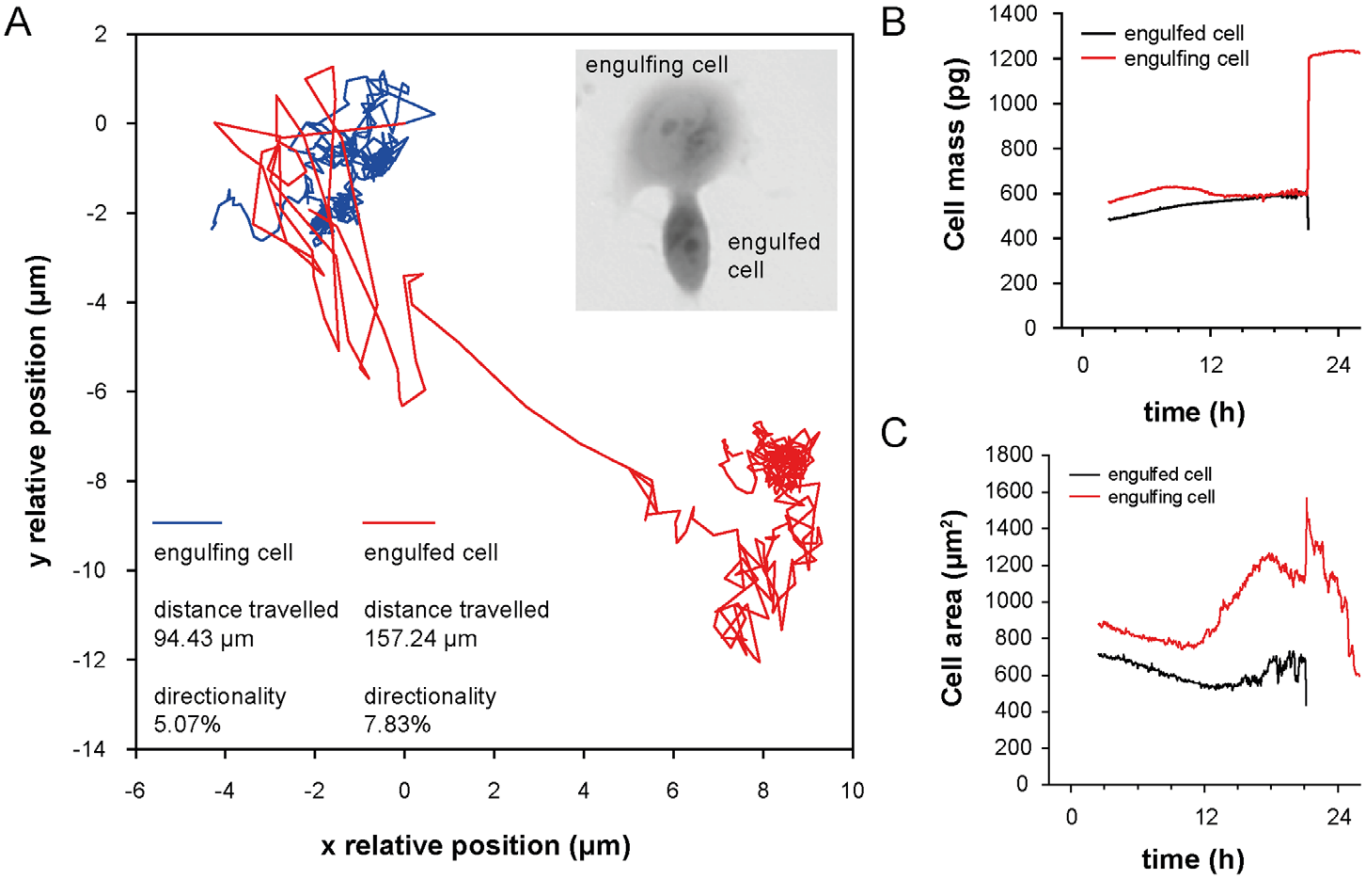
Mechanistic characterization of engulfed and engulfing cells in entosis. **(A)** Trajectory travelled of both engulfing and engulfed cell until cell fusion. See differences in the travelled distance and in directionality of individual cells. Directionality describes "purposefulness" of the movement where 0% indicate random movement and 100% indicate straight line trajectory between starting and ending position. Position (0.0, 0.0) indicate place of cell fusion. **(B)** Changes in cell mass and **(C)** cell area of engulfed and engulfing cell.

Moreover, we observed entosis triggering 20 h after the plumbagin treatment in the tumour cell population exposed to plumbagin. During entosis, cells invaded neighbouring cells, which led to the formation of cell-in-cell structure (see Fig 3A, S1 Video), no bird eye structure was observed. Internalized cells played an active role in their engulfment (see Fig 4), which resulted in complete internalization. Cells which have undergone entosis (both engulfing and even engulfed cells) lived about five hours longer than the other observed tumour cells (frequency of this phenomenon was 2.5%). After 48 h of treatment, all PC-3 cells observed by holographic-microscopy were dead.

### Time-lapse flow-cytometry

To evaluate the amount of cells positive or negative for typical markers, such as phosphatidyl serine exposure, presence of autophagosomes, or intact cellular membrane and nuclear DNA content, we used flow-cytometry at 12 time points (0h, 40min, 1.5h, 4h, 6h, 8h, 10h, 16h, 20h, 24h, 36h, and 48h). Phosphatidyl serine exposure was detected by Annexin V staining, cell viability by propidium iodide (PI) and SYTO 16 staining, and CYTO-ID Green was used as a marker of autophagosome formation. To determine the proportion of large cells with intact DNA, we used SYTO 16 and FSC. At the beginning of the experiment, about 30% of all cells were large with an intensive SYTO 16++ signal (Fig 1E). A decrease in the amount of SYTO 16++ large cells was shown during the first 8 hours of plumbagin treatment (ca. 15% SYTO 16++ large cells) and an increase in the amount of SYTO 16++ large cells were shown after 10h of treatment (about 20%). As we observed no dividing cells during the treatment (see S1 Video), emergence of giant cancer cells (PGCCs) larger than the average population could be assumed namely because no increase in the percentage of annexin V-/PI- cells (healthy cells that would result from dividing) was observed around the 10h time-point of treatment (Fig 1E).

**Fig 5:**
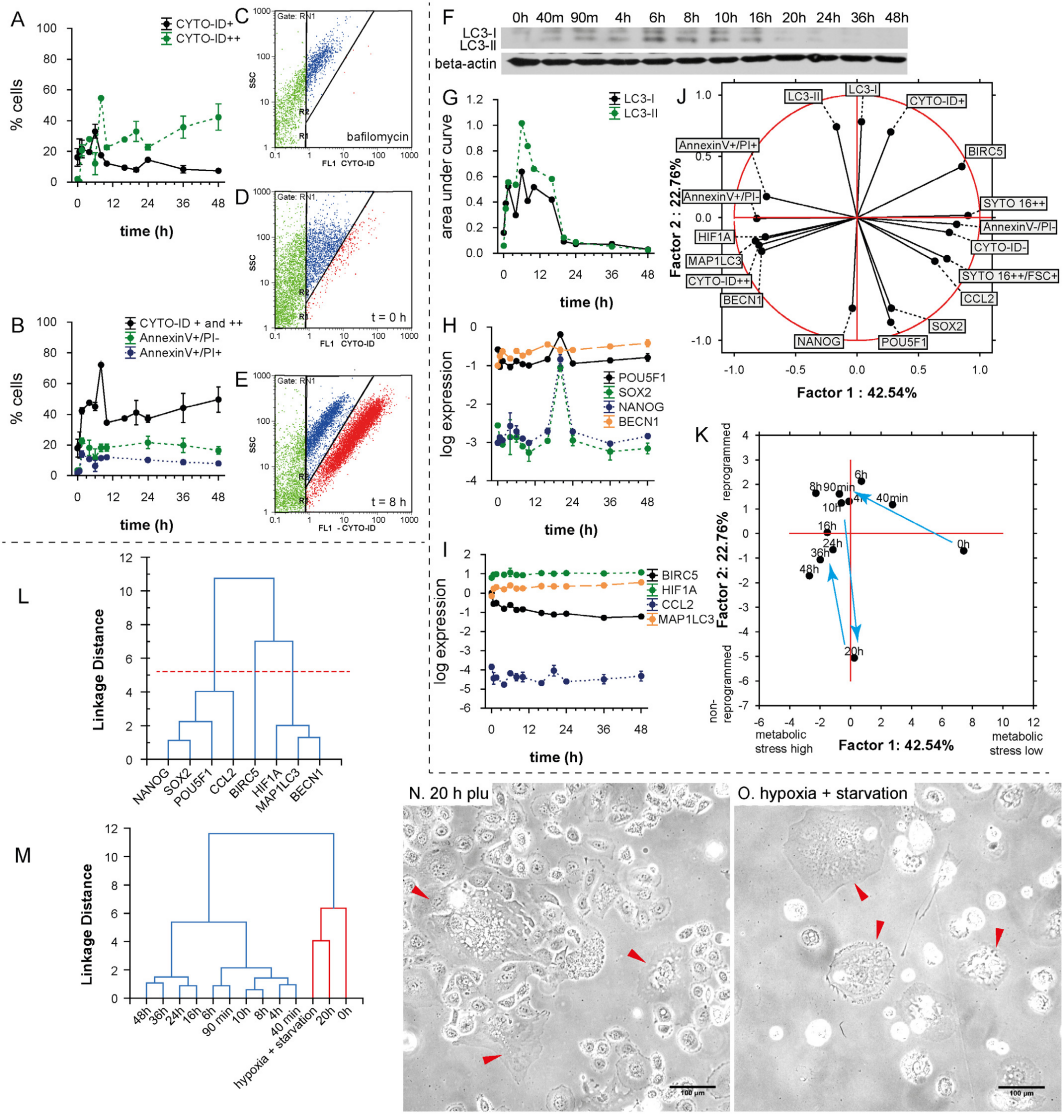
Autophagy and self-renewal after plumbagin treatment. **(A)** Time dependent dynamics of CYTO-ID Green (CYTO-ID+, CYTO-ID++) after 2 μM plumbagin treatment. See autophagy peak at 8h-time point. **(B)** Time dependent dynamics of cell deaths after 2 μM plumbagin treatment; total CytoID staining (CYTO-ID+ and CYTO-ID++) depicts autophagy, AnnexinV+/PI- depicts apoptosis or early oncosis, and AnnexinV+/PI+ depicts necrosis (raw data and gating strategy in S2 Appendix). **(C)** Amount of autophagic cells after bafilomycin treatment; red cluster depicts CYTO-ID++ population, blue cluster Cyto-ID+ population. **(D)** Amount of autophagic cells in control (not-treated population); red cluster depicts CYTO-ID++ population, blue cluster CYTO-ID+ population. **(E)** Amount of autophagic cells after plumbagin treatment; red cluster depicts CYTO-ID++ population, blue cluster CYTO-ID+ population. **(F)** Western blot for LC3-I and LC3-II isoforms at 12 time-points after 2 μM plumbagin treatment (raw data in S3 Appendix). **(G)** Graphic representation of western blot results for LC3-I and LC3-II isoforms at 12 time-points after 2 μM plumbagin treatment. **(H)** Time dependent dynamics of *POU5F*, *SOX2*, *NANOG*, and *BECN1* gene expression after 2 μM plumbagin treatment. **(I)** Time dependent dynamics of *BIRC5*, *HIF1A*, *CCL2*, and *MAP1LC3* gene expression after 2 μM plumbagin treatments. **(J)** Principal component analysis—projection of variables on the two-factor plane. See distinct clustering of genes with flow-cytometric measurements based on metabolic stress and reprogramming (for details see results). **(K)** Principal component analysis—projection of time-points on the two-factor plane. The first and the second factor are designated as “metabolic stress high-low” and “non-reprogrammed-reprogrammed”, respectively (for details see results). **(L)** Cluster analysis of gene expression. The “reprogramming cluster”involved *POU5F*, *SOX2*, *NANOG*, and *CCL2*; the “autophagic and hypoxia cluster”involved *HIF1A*, *MAP1LC3*, and *BECN1*; the third cluster involved *BIRC5* gene. **(M)** Cluster analysis of gene expression of 12 time-points after 2 μM plumbagin treatment and PGCCs selected by hypoxia and starvation; based on correlations of gene expression patterns, similarity between PGCCs and PC-3 cells after 20h plumbagin treatment was found. **(N)** Morphology of cells after 20h plumbagin treatment; cells with polyploid giant cancer cell (PGCCs)-like morphology are highlighted by arrows. **(O)** Morphology of PC-3 cells after 1 month of hypoxia and starvation; cells with PGCCs-like morphology are highlighted by arrows.

Furthermore, we used the CYTO-ID Green autophagy dye for autophagy detection. Previously, CYTO-ID was validated by observing co-localization of the dye and RFP-LC3 in HeLa cells using fluorescence microscopy. An increase in CYTO-ID signal indicates the accumulation of autophagosomes [31]. Nevertheless, we detected two cell populations according to the intensity of CYTO-ID signal (CYTO-ID+, CYTO-ID++) (Fig 5A and 5E). The CYTO-ID+ population correlated with the LC3-I form assessed by western-blotting (Fig 5F and 5G) (r = 0.66; p = 0.001) and the CYTO-ID++ population correlated with the LC3-II/LC3-I ratio (r = 0.49; p = 0.016). A correlation between total CYTO-ID staining and the LC3-II/LC3-I ratio was found, too (r = 0.70; p = 0.0001). According to our observation, plumbagin functions as an inductor of autophagy in the context of PC-3 cells. We also performed the flow-cytometric CYTO-ID analysis of non-treated PC-3 (Fig 5D), and plumbagin (Fig 5E) and bafilomycin (Fig 5C) (inhibitor of autophagy) treatments. During the plumbagin treatment, a high positive peak of CYTO-ID++ was shown at the time point of 8h. After the 8h peak, signal diminution was observed (probably LC3-II deconjugation and partial degradation of acid autophagic vacuoles); see Fig 5A. A decrease in the CYTO-ID++ signal after the time-point of 8h indicates that there is not only the simple accumulation of autophagosomes, but also the degradation of their content. During the whole experiment (48 h), the level of CYTO-ID++ signal was higher than at the zero time-point.

The highest percentage of autophagic cells (according to the accumulation of autophagosomes) was observed 8 hours after the treatment; see Fig 5B. Due to the triggering of autophagic mechanisms, almost no increase of necrotic (AnnexinV+/PI+) and early oncotic or apoptotic (AnnexinV+/PI-) cells was induced (Fig 5B). Almost 40% of cells were autophagic during the 48 h experiment. The quantity of AnnexinV+/PI+ and AnnexinV+/PI- did not exceed 20% at any observed time-point.

### Time-lapse gene expression profiling

We isolated RNA (in duplicates) from the PC-3 cells at 12 time-points (0h, 40min, 1.5h, 4h, 6h, 8h, 10h, 16h, 20h, 24h, 36h, and 48h) of plumbagin treatment and performed qRT-PCR. Expression was assessed of *BECN1*, *MAP1LC3A*, *HIF1A*, *BIRC*, *CCL2*, *POU5F1*, *SOX2*, and *NANOG*. During the experiment, we observed a gradual increase in the expression of autophagy-related genes *MAP1LC3A* and *BECN1* and a gradual decrease of *BIRC*. The expression of autophagy-related genes was in a good correlation (r = 0.92, p<0.001) and correlated also with *HIF1A* (r = 0.87, p<0.001 and r = 0.79, p<0.001); see Fig 5L. We also found an extensive induction of pluripotency-associated genes expression (*NANOG*, *SOX2*, and *POU5F1*) at the time-point of 20 h; see Fig 5H. No dramatic changes in *HIF1A* expression were observed during the 12 time-points measurements (Fig 5I). Among the analyzed genes, three different expression clusters were found (see Fig 5L). The most expressed gene cluster was autophagic promoting cluster of genes (*BECN1*, *MAP1LC3A*, and *HIF1A)*. Of all the observed genes, the least expressed one was *CCL2* (Fig 5I).

### Principal component analysis

In addition to the correlation analysis, the component analysis made it possible for us to detect the structure of relationships between the observed time-points, thus helping us to assess main variables in the particular time-points. Moreover, this analysis allowed us to classify variables based on the flow-cytometric and expression profiles. To illustrate the model of metabolic stress and self-renewal, two-factor analysis was chosen with a total cumulative variance of 65.3% and eigenvalues 42.54% and 22.76% for factor 1 and factor 2, respectively (Fig 5J). This two-axis model represents both metabolic stress (factor 1) and “self-renewal and reprogramming capacity” (factor 2). These conclusions are based on the following findings: (a) *NANOG*, *SOX2*, and *POU5F1* are genes, which strongly correlate with the pluripotency and self-renewal, (b) annexinV+/PI+ status refers to cell death; (c) PI positivity, Annexin V positivity, CYTO-ID positivity, *BECN1*, *MAP1LC3A*, and *HIF1A* expression are directed to the negative values of factor 1 (high metabolic stress). Taken together, positive values of factor 1 are associated with the healthy cells rather than with the cell damage, and negative values of factor 2 are associated with cell reprogramming processes, rather than with the naive PC-3 cells. When we accept this model, large cells with the intensive SYTO 16++ signal seem to be further in reprogramming processes than the average SYTO 16++ population of PC-3 cells and naive PC-3 harvested at the zero time-point, and *BIRC* expression is very important for preventing metabolic stress in the naive PC-3, but could also prevent reprogramming mechanisms.

When these factors are used to project cases (time-points), another apparent trend is evident: At the time-point of 20h, PC-3 cells seem to break free from the bondage of metabolic stress by triggering the reprogramming processes. This “reprogrammed” PC-3, are associated with the lower metabolic stress (as represented by positive values of factor 1). However, when the stress continues, the cells are being pushed to higher metabolic stress again, staying partially reprogrammed though; see Fig 5K time-point 48 h. We could assume some cyclic characteristic of stress resistance processes and reprogramming. During these cycles of adaptation, the cells are pushed to a state that is increasingly dedifferentiated.

### Polyploid giant cancer cells (PGCCs) expression profiling

When the PC-3 cancer cell line was cultured under normal conditions, some large cells with enlarged nuclei (PGCCs) were sporadically observed. However, the exposure of PC-3 cancer cells to long-term starvation and hypoxia (for at least one month) induced the formation of cells with larger cell and nuclear size, while cells of normal morphology were almost eliminated. PGCCs were highly resistant to oxygen deprivation and could generate regular-sized cancer cells through budding or bursting. The PGCCs induced by starvation and hypoxia were 2–10 times larger than normal cells, and had a distinctive morphology (see Fig 5O).

Next, qRT-PCR gene expression profiling was performed to analyze the specific molecular pattern expressed in PGCCs. Expression profiles of eight genes (*BECN1*, *MAP1LC3A*, *HIF1A*, *BIRC*, *CCL2*, *POU5F1*, *SOX2*, and *NANOG*) were assessed and compared with the normal PC-3 cell expression patterns obtained at the particular time points during the plumbagin treatment by cluster analysis (see Fig 5M). After 20h of plumbagin treatment, the expression pattern of PC-3 cells matched with the PGCCs characteristics as well as the cell morphology (see Fig 5N and 5O).

## Discussion

Panov *et al*. found out, that the PC-3 prostate cancer cell line contained 2 to 4 times more mitochondria with enhanced respiratory activity [15], which may cause high sensitivity of PC-3 cells to ROS—produced massively during the plumbagin treatment. However, in cytotoxicity tests we have noticed differences in sensitivity to plumbagin measured by metabolic-based MTT and impedance-based RTCA. The output of RTCA method is a cell index value (CI; see Fig 1A) that reflects the number of cells as well as morphological parameters such as size, shape, and degree of cell attachment to the substrate. This means that an increase in the average size of surviving cells affects CI and could correlate with higher IC50 values (1.4 μM resp. 10 μM IC50 after 6h resp. 24h treatment) As we observed no dividing cells during the 2 μM plumbagin treatment (S1 Video), we assumed that the cells increased their volume between the time-points of 6h and 24h. Accordingly, an increase in the amount of SYTO 16++ large cells was shown at the 20h time-point as compared with the 6h time-point (see Fig 1F and 1I). This might indicate the emergence of giant polyploid cancer cells. SYTO 16 is effective at staining the DNA of cells, because it binds preferentially to DNA over RNA at a ratio of approximately 20:1 [32] and shows specific information about the cell nuclei, such as size and distribution. Furthermore, Manusco *et al*. used SYTO16 staining to enumerate circulating endothelial cells with a high DNA content [33] and Ibrahim *et al*. identified multinucleate cells during the growth cycle of *Mycobacterium avium* by SYTO 16 staining [34]. Importantly, Wlodkowic *et al*. have recently showed that SYTO dyes do not adversely affect normal cellular physiology. The cytometric detection of SYTO 16 fluorescence loss is even a sensitive marker of early apoptotic events [35].

Unlike the RTCA IC50 values, these values measured by MTT were relatively stable (2.66 μM resp. 2.42 μM IC50 after 6h resp. 24h treatment). The main mechanism of MTT assay is a reduction of tetrazolium salts to formazan by mitochondrial succinate dehydrogenase (SDH). SDH loses its activity by damage of the respiratory chain [36]. According to our results, main reduction of SDH activity takes place during the first 6 hours. As mitochondria are the main producer of ROS, they are an easy target of ROS-mediated damage, too. Thus, the ROS-mediated damage plays a key role in the induction of cellular senescence [37]. In this study we disclosed that the major ROS producing mitochondria in the PC-3 cells are coated by ER membranes. Since the ER mitochondria encounter structure (ERMES) plays a key role in mitophagy in yeast and Boeckler *et al*. showed that successful mitophagy depends on mitochondrial ER tethering, [38], we assume that the major ROS producers in PC-3 are destined for degradation by mitophagy. Mitophagy helps to eliminate damaged mitochondria and maintains their healthy pool [39]. Mitophagy also presents a possible way to gain enough energy for survival [19] and simultaneously removes the ROS producing mitochondria [40]. Accordingly, PC-3 cells express a high amount of pro-mitophagic genes *PINK1*, *FUNDC1*, and *SMURF1* in comparison with the other cell lines of NCI60 panel. On the other hand, *PARL* (responsible for PINK1 cleavage) was underexpressed. The PINK1 protein is connected with the mitochondrial quality control by targeting damaged mitochondria for degradation [41]. The loss of PINK1 also impairs stress-induced autophagy and cell survival [42]. Furthermore, the mitochondrial outer-membrane protein FUNDC1 could mediate hypoxia-induced mitophagy [43]. According to these data, it seems possible that PC-3 cells have a high level of mitochondrial quality control and are able to identify and then degrade the damaged mitochondria. It could seem that getting rid of mitochondria is not much favourable for the tumour cell in the long run, because tumour cells without mitochondrial DNA (mtDNA) show retarded tumour growth [44]. Nevertheless, the tumour formation could be associated with the acquisition of mtDNA from host cells [44] and this temporary handicap could be thus compensated.

Severely damaged cells often exhibit accumulation of autophagosomes and hence seem to be subject to autophagic cell death. Nevertheless, in many cases, this “autophagic cell death” is the cell death during autophagy rather than the cell death by autophagy [45]. In our study, the highest percentage of autophagic cells was observed 8 hours after the treatment (see Fig 5B). Due to the triggering of autophagic mechanisms, almost no increase of necrotic (AnnexinV+/PI+) and early oncotic or apoptotic (AnnexinV+/PI-) cells was induced. It follows that induction of autophagy does not seem to be directly related to cell death. Conversely, our results suggest that autophagy can promote the survival of cells under oxidative stress, which is in accordance with the results of other studies [24, 46]. According to our results, autophagy precedes several mechanisms such as self-renewal and entosis. Sun *et al*. found out that tumour cells with high deformability preferentially engulf neighbouring cells with low deformability in heterogeneous populations. They also found out that downregulation of contractile myosin allows the internalization of neighbouring cells and that a mechanical differential between the engulfing and engulfed cells is required for entosis to progress [47]. Therefore, we would like to present a hypothesis, that reduction in membrane and cell stiffness due to protein catabolism by autophagy could reflect increased entotic activity. Moreover, the entotic vacuole membrane encircling the internalized cells recruits the autophagy protein LC-3 [48]. The initiation of entosis, instead of apoptosis or necrosis, might give the cell additional time to survive the transient deleterious conditions. The cell in cell structure results in the decreased surface-to-volume ratio, thereby minimizing cell membrane requirements (one membrane for two nuclei) (Fig 4). Furthermore, a live cell internalized by entosis could disrupt host cell division. Subsequently, cytokinesis often fails, which causes the formation of binucleate cells that are able to generate aneuploid cell lineages [49]. It was also shown that the frequency of entotic structures correlates well with the tumour grade [49]. According to this observation, we speculate, that entosis and competition between cells by cannibalism is rather late-stage process in cancer cells and requires activation of mechanisms leading to higher developmental plasticity, changes in cytoskeletal proteins [47] and some kind of de-differentiation of cells accompanied with changes in transcription regulators. All of these could be supported by ROS. For example, transcription factor C/EBPbeta (LIP), is elevated by endoplasmic reticulum stress and oncogenic signalling [50, 51]. Recently, expression of LIP was connected with enhanced autophagy and engulfment of neighbouring cells in the human breast cancer cell line (MDA-MB-468 cell line with mutant p53 [52]) [53]. In our experiment, entotic cells lived by about five hours longer than the other observed tumour cells. Although the all observed PC-3 cells were dead after 48h treatment, it could probably be only the consequence of relatively low numbers of cells (ca. 50 cells) which can be seen in the holographic-microscope field of view. Nevertheless, according to the flow-cytometry results, more than 60% of cells were annexinV-/PI- after 48 h of 2 μM plumbagin treatment. About 18% of these cells were large and SYTO 16++.

Similar to mitophagy, digestion of the cytoplasm of neighbouring cells can provide a source of amino acids, indicating that cannibalism could be a survival mechanism for tumour cells during starvation and other adverse conditions [54]. Significant reduction of cell motility and cannibalism were found after the plumbagin treatment. Furthermore, plumbagin is able to suppress activation of nuclear factor-κB (NF-κB) [13], and therefore could repress anoikis resistance [55], which may be an explanation why plumbagin is probably more toxic for the PC-3 cell line than cisplatin (2.42 μM vs. 75 μM IC50 value after 24h treatment) [8].

We have also found out that specific processes leading to induction of reprogramming to pluripotency (depicted by significant overexpression of *NANOG*, *SOX2*, and *POU5F1* [17, 26, 56]) were triggered 20h after the treatment in the tumour cell population exposed to ROS. These three pluripotency-related transcription factors, Oct 3/4, Nanog, and Sox-2, form a core regulatory network that coordinates the self-renewal and differentiation of stem cells. Transfection of NANOG, SOX2, OCT4, and LIN28 in human fibroblasts induced pluripotency, indicating a key position of these factors in the reprogramming of somatic cells [57]. These self-renewal molecules highly contribute to tumourigenesis [17, 58]. Takahashi *et al*. also showed that the introduction of transcription factors Oct3/4, Sox2, c-Myc, and Klf4 into mouse adult fibroblasts was able to reprogramme differentiated cells to an embryonic-like, pluripotent state [59, 60]. Nanog overexpression was found to be one of the distinctive features of the population of fibroblasts that escaped from Ras-induced senescence [61]. Furthermore, Nanog was found to be promoting the transfer of pluripotency after the cell fusion of reprogrammed and non-reprogrammed cell [62]. Nanog seems to be required during the final stages of somatic cell reprogramming. Once the pluripotent state is established, Nanog is no longer needed [63], which is in accordance with the peak-character of *NANOG* expression observed by us (see Fig 5H). Bambrik *et al*. postulated that the activation of endogenous Oct4 or Nanog may be a marker for fully reprogrammed induced pluripotent stem cells (iPSCs) [64]. We rather suppose, that overexpression of *NANOG*, *SOX2*, and *POU5F* in the prostate tumour cell population (especially in PGCCs; [65]) exposed to ROS leads to higher developmental plasticity and capability to faster respond to changes in the extracellular environment that could ultimately lead to alteration of cell fate (epithelial features vs. mesenchymal character etc.). Similar process was observed in Mitchell et al. study [66]. Expression of pluripotency genes results in functional reprogramming that could provide to tumour cells higher developmental potency [67] as well as higher stemness [25]. For example, it was observed that differentiated cancer cells and tumor stroma can be originated directly from polyploid giant cancer cells induced by paclitaxel [67].

**Fig 6:**
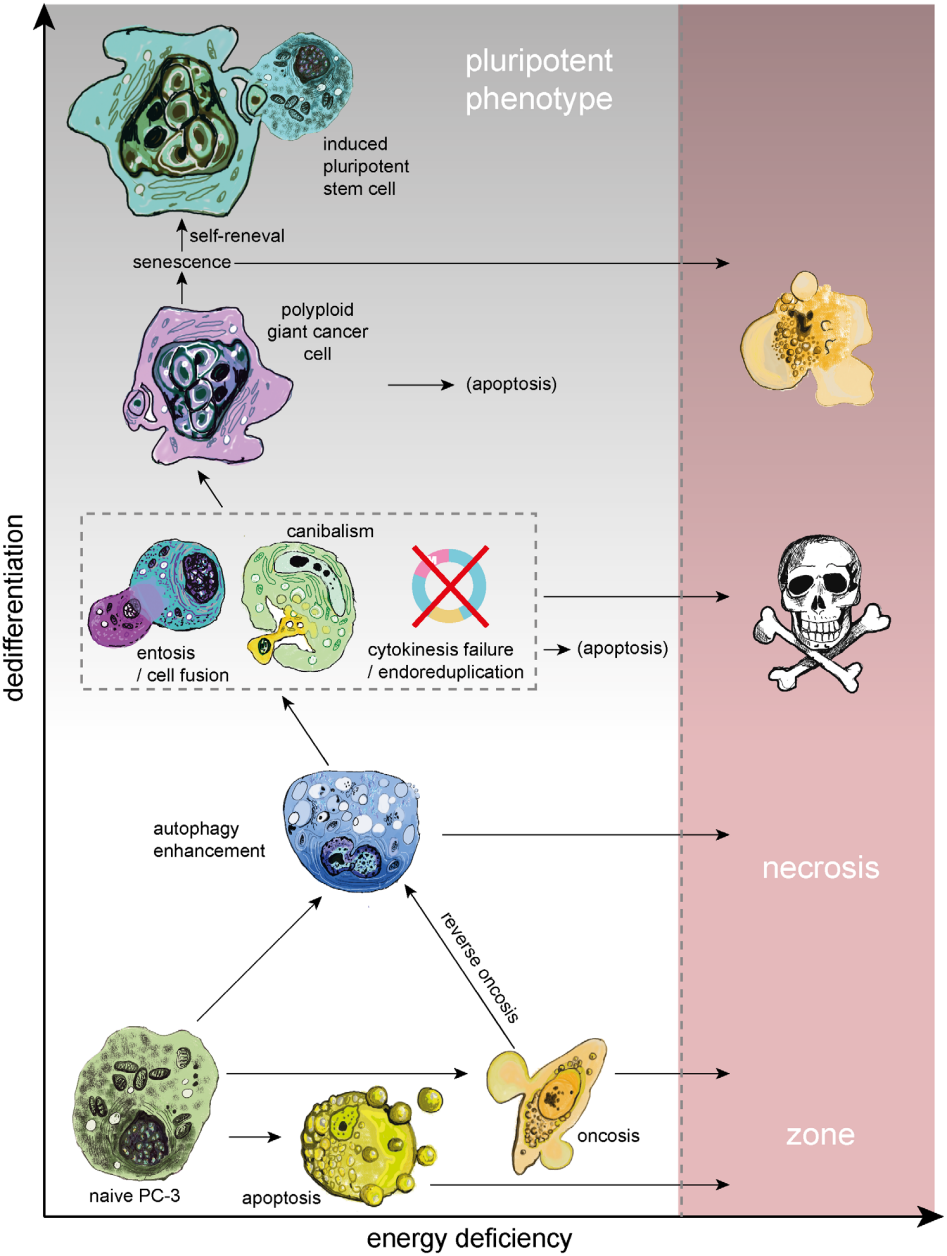
Possible cell fate under oxidative stress. Prolonged oxidative stress (ROS) leads to severe cell damage and depletion of cell energy reserves. Nevertheless, many cell injuries caused by ROS could be sublethal, especially because PC-3 cells have non-functional p53 and therefore disrupt the triggering of apoptosis. Even if a damaged cell is driven to oncosis, reversion of this process is possible, particularly if the cell is able to restore ATP production. A way to gain enough energy for survival could be autophagy. Similar to autophagy, digestion of the cytoplasm of neighbouring cells can provide a source of amino acids. Retention of a foreign nucleic acid by cannibalistic engulfment could result in aneuploid or polyploid state. Furthermore, reduction in membrane and cell stiffness due to protein catabolism by autophagy could reflect increased entotic activity. Cell in cell structure results in the decreased surface-to-volume ratio, thereby minimizing cell membrane requirements. Furthermore, a live cell internalized by entosis could disrupt host cell division. Subsequently, cytokinesis often fails, which can lead to the formation of polyploid giant cancer cells (PGCCs). PGCCs often dye by apoptosis or senescence, but a small fraction of these cells is able to survive and even produce aneuploid progeny. Senescence, polyploidy and self-renewal seem to be three steps to immortality of cancer cells. Autophagy could play an important role in all of them.

Here, we hypothesize that autophagy and cell-in-cell structures accompanied with polyploidization could also play an important role in the survival, remodelling and dedifferentiation of cells to a pluripotent state (for summary see Fig 6). Autophagy could cause the degradation of proteins important for the status of differentiation, which could restrain the process of reprogramming, and simultaneously could eliminate proteins that should not be expressed in the pluripotent cells. According to our model arisen from the principal component analysis, *BIRC* expression is very important for preventing metabolic stress in naive PC-3, but could prevent reprogramming mechanisms, too. Protein product of *BIRC* gene (survivin) is able to prevent apoptotic cell death, but also inhibits autophagy [68, 69], which could contribute to the efficiency of puripotent stem cells generation [26]. Furthermore, relatively little expressed in PC-3 cells is another autophagy inhibitor *CCL2* [69].

In our experiment, the expression pattern of PC-3 cells as well as their morphology after 20h of plumbagin treatment matched with the PGCCs characteristics. This observation is in accordance with the idea that entering polyploidy is part of a strictly regulated process that could provide a survival advantage to cells with DNA damage [17]. Notably, the polyploid state can affect the resistance of cancer cells and could also contribute to the generation of cancer stem cells in response to stress [17, 25]. According to our model, large cells with an intensive SYTO 16++ signal (probably PGCCs) seem to be further in reprogramming to pluripotency processes than naive PC-3 cells. Erenpreisa *et al*. discussed, that senescence, polyploidy and self-renewal are three steps to immortality of cancer cells [17]. We predict that autophagy could play an important role in all these three processes, particularly because PC-3 are p53 deficient and hence could be resistant to senescence, which is a natural barrier for reprogramming [26]. PC-3 seem to be predisposed to reprogramming [70–72].

We have already tested other prostate cell lines such as PNT1A and 22Rv1, but they both have partially active p53 and therefore they are not resistant to cell cycle arrest, senescence and cell death [8] triggered due to polyploidization. It is in accordance with other studies where initiation and maintenance of senescence seems to be p53 dependent [37, 73] as well as cell death triggering after polyploidization [74]. According to these results, we supposed, that polyploidization and PGCCs forming as mechanism of resistance is rather typical for cell lines that have p53-deficiency and metastatic potential and is not unique for prostate cancer cell lines. Recent work by Zhang et al. has confirmed the importance of PGCCs in therapy resistance and ovarian cancer progression [25, 75]. In contrast with polyploidization, we supposed that mitophagy is more general principle of coping with cell stress in immortalized cells, which is in accordance with other studies [76–78].

Prostate cancers often show large intra-tumour heterogeneity in almost all measurable characteristics including metabolism, gene expression, cellular morphology, and metastatic potential. Some cell populations inside the tumour seem to be predisposed to resist high levels of metabolic stress. One of the ways how to resist is proper control and degradation of damaged ROS producing mitochondria by mitophagy. Accordingly, autophagy was activated as a protective mechanism and mediated the resistance phenotype of some cancer cells during the ROS-producing treatment in our model of apoptosis-resistant, androgen-independent, metastasis producing prostate cancer (PC-3 cell line). In recent years, it seemed possible that autophagy can execute cell death in cancer cells which are apoptosis defective, but this is not true for the PC-3 cell line. We also assume that autophagy could promote arousal of cell-in-cell structures and thus play a significant role in polyploidization and dedifferentiation of cells to a pluripotent state. Inasmuch as androgen-independent prostate cancer cells with non-functional p53 are able to quite easy cope with damages caused by ROS producing agents and ROS can even participate in positive selection of resistant PGCCs with some pluripotent characteristics, prostate cancer therapies based on ROS producing agents could be a misleading concept.

## Supporting Information

S1 Appendix Expression of mitophagy-related genes by NCI60 platform. For the assessment of the relative intensity of mitophagy related genes expression (*PINK1*, *FUNDC1*, *SMURF1*, and *PARL*) in the PC3 cell line we used CellMiner Database (http://discover.nci.nih.gov/cellminer/). It allows the determination of selected genes expression patterns from 5 microarray platforms in 60 cell lines (NCI60 panel). The tool output includes relative transcript intensity presented as z-scores and visualized as a bar graph. The bars for each cell line are colour-coded by the tissue of origin.
(TIF)

S2 Appendix Flow-cytometric analyses. Gating strategy of time-lapse analysis of plumbagin-treated cells using AnnexinV/Propidium iodide, SYTO 16 and CYTO-ID staining and raw data.
(PDF)

S3 Appendix Western blotting of LC-3. The cropped part of the blot identical to Fig 4 and whole membrane with LC-3 and beta-actin antibodies.
(TIF)

S1 Video Time-lapse imaging of entosis and oncosis. 2μM plumbagin treatment. **No cell division** appears during 48 hour of plumbagin treatment; red square areas highlight main points of interest; A) during entosis, internalized cell plays an active role in process of internalization. Both, engulfing and engulfed cell live about five hours longer than the other PC-3 cells. B) Oncotic cells formed cytoplasmic blebs and showed chromatin clumping followed by necrotic features such as cell membrane rupture and detaching from the surface.
(MP4)

S2 Video Time-lapse imaging of cannibalism with the cell fusion (digestion of engulfed cell, detail). Target-cell is gradually engulfed by active cannibalic polynuclear cell. Bird eye structure typical for cannibalism appears. Then the target cell dies off.
(MP4)

S3 Video Time-lapse imaging of cannibalism without the cell fusion (detail). Weakened target- cell is contacted by cannibalic cell, exploited and left to die.
(MP4)

S4 Video Time-lapse imaging of reverse oncosis (detail). Recuperation of oncotic cell forming cytoplasmic blebs.
(MP4)

S5 Video Time-lapse imaging of untreated PC-3 cell line. Unaffected PC-3 cells. Red arrows depict cell division.
(MP4)
