# Supplementary figures and images for: Oxidative Stress Resistance in Metastatic Prostate Cancer: Renewal by Self-Eating
Source: PLoS One. 2015 Dec 15;10(12):e0145016. doi: 10.1371/journal.pone.0145016 (PMC4679176; doi:10.1371/journal.pone.0145016)

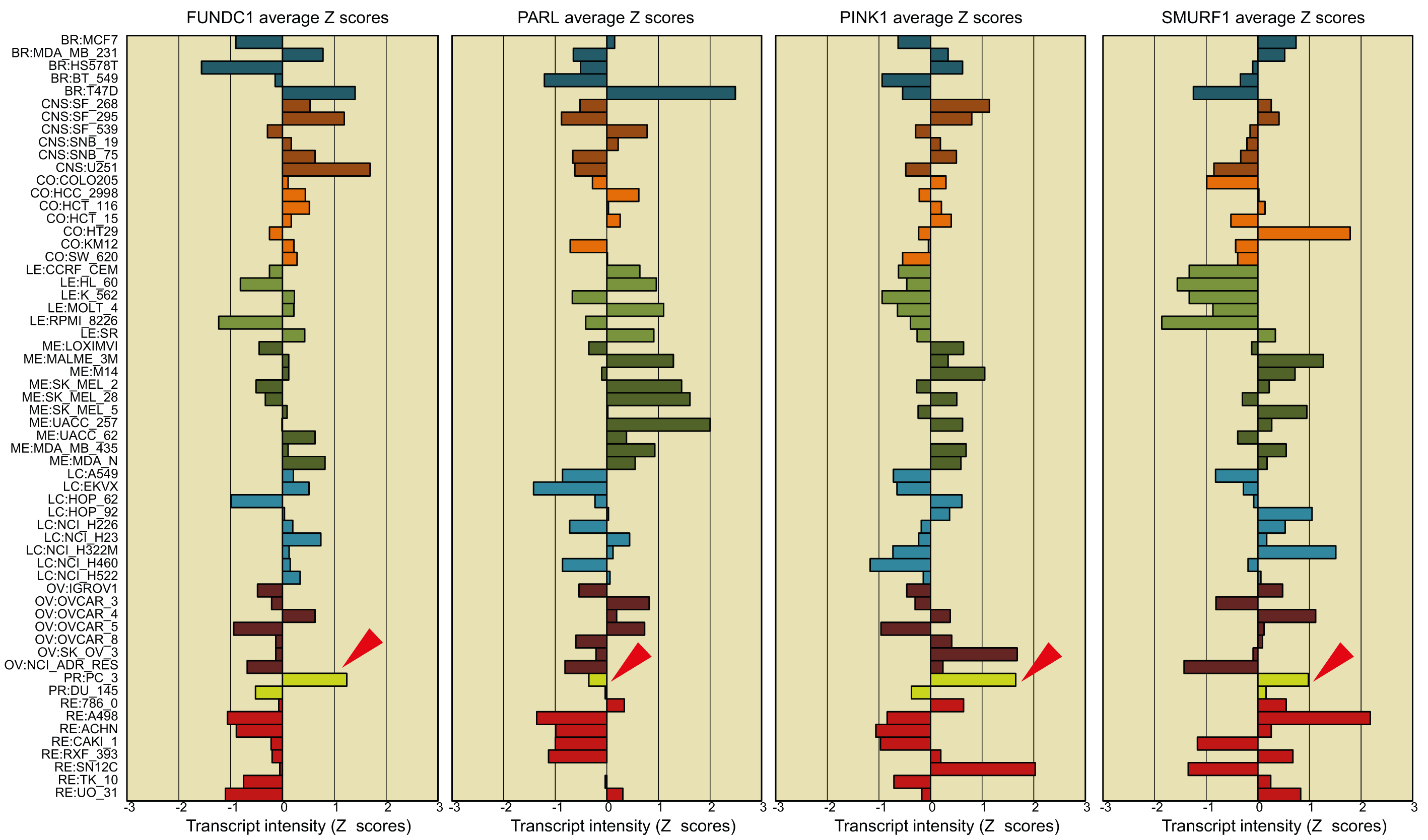

Supplement: S1 Appendix — For the assessment of the relative intensity of mitophagy related genes expression (PINK1, FUNDC1, SMURF1, and PARL) in the PC3 cell line we used CellMiner Database (http://discover.nci.nih.gov/cellminer/). It allows the determination of selected genes expression patterns from 5 microarray platforms in 60 cell lines (NCI60 panel). The tool output includes relative transcript intensity presented as z-scores and visualized as a bar graph. The bars for each cell line are colour-coded by the tissue of origin. (TIF) [file pone.0145016.s001.tif]

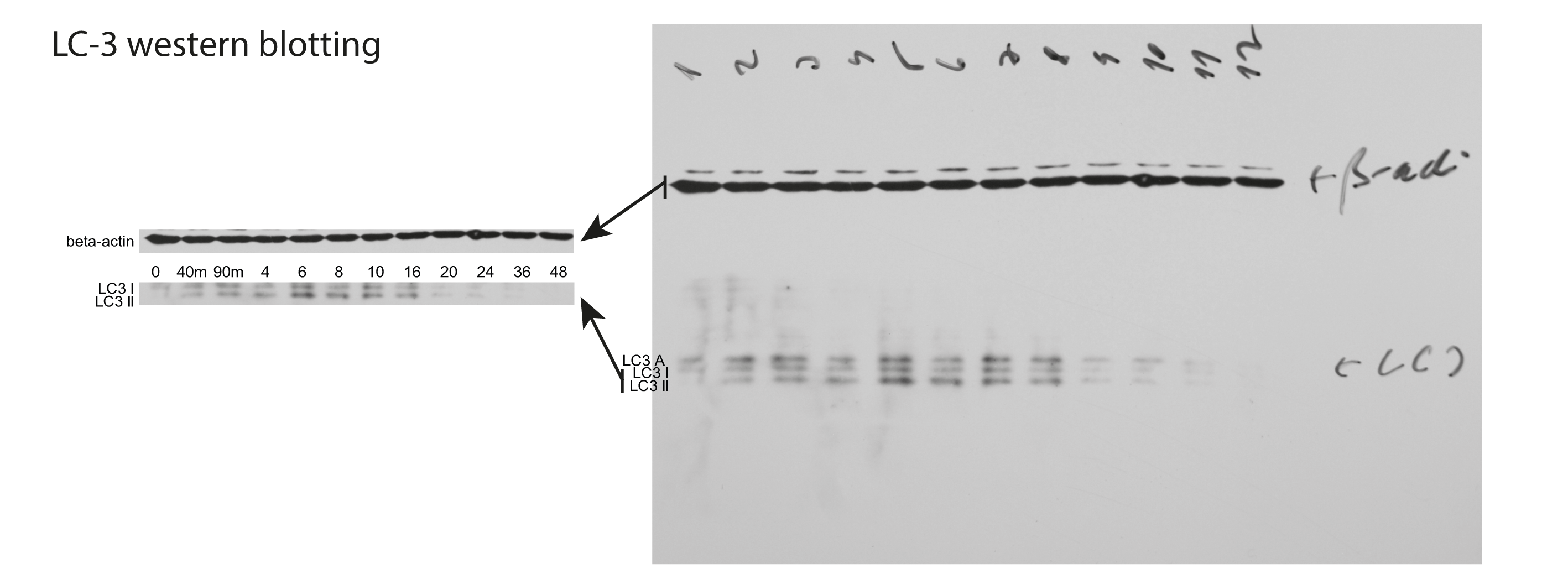

Supplement: S3 Appendix — The cropped part of the blot identical to Fig 4 and whole membrane with LC-3 and beta-actin antibodies. (TIF) [file pone.0145016.s003.tif]
