# Supplementary material for: Oxidative Stress Resistance in Metastatic Prostate Cancer: Renewal by Self-Eating
Source: PLoS One. 2015 Dec 15;10(12):e0145016. doi: 10.1371/journal.pone.0145016 (PMC4679176; doi:10.1371/journal.pone.0145016)
Supplement: S2 Appendix — Gating strategy of time-lapse analysis of plumbagin-treated cells using AnnexinV/Propidium iodide, SYTO 16 and CYTO-ID staining and raw data. (PDF) [file pone.0145016.s002.pdf]

Flow-cytometric analysis - raw data and gating strategy

Gating strategy

In all flow cytometric gating strategies we used defined range (RN1) for debris elimination.

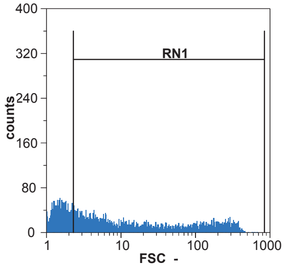

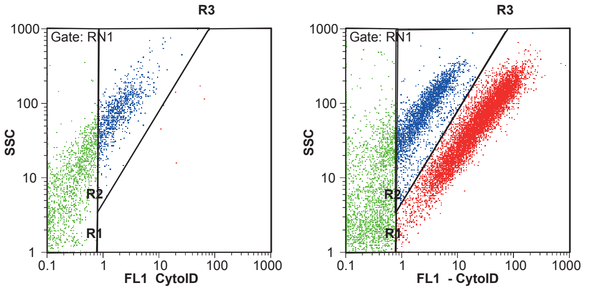

The CytoID autophagy detection kit is based on the determination of cells positive for the presence of acidic organelles co-localized with LC-3b protein. For negative control we used bafilomycin A1 treated cells. Bafilomycin A1 prevents organelles acidification by inhibiting vacuolar H+ ATPase. CytoID positive cells were divided into two populations, based on the signal strength (red cluster depicts CYTO-ID++ population, blue cluster Cyto-ID+ population). Weak Cyto-ID-positivity is presented even after Bafilomycin A1 treatment due to residual activity of acidic organelles (blue cluster).

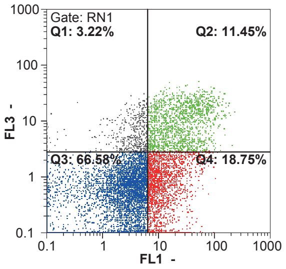

For the determination of “normal” (“healthy”), “early apoptotic”, “late apoptotic/necrotic” cells and “cellular fragments”, classical four quadrant gating strategy was performed.

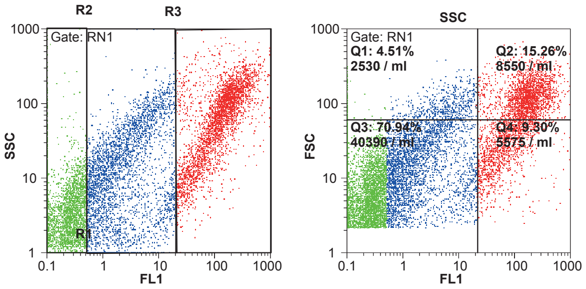

For the determination of large, SYTO16 highly positive cells, two gating strategies were applied. In first strategy (SSC/-FL1), cells were divided into three population according their positivity for SYTO 16 staining (negative/slightly positive/highly positive), and granularity. After that, second gating strategy (FSC/FL1) was performed for determining of SYTO16 positive-large sized population of cells.

Flow-cytometric analysis - CytoID staining - plumbagin-treated PC3 cells

Negative control - Bafilomycin treatment

File: autof_BAFILOMYCIN.FCS Date: 20-02-2015 Time: 13:06:37 Particles: 5000 Acq.-Time: 178 s partec PAS

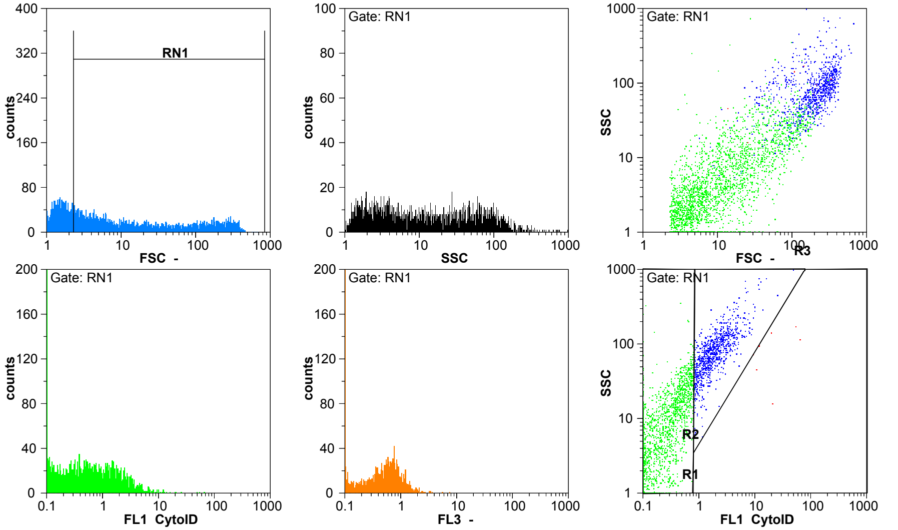

R3: 0.14% R1: 82.16% R2: 17.90%

Region	Gate	Count	Count/ml	%Gated	Mean-x	CV-x%	Mean-y	CV-y%
R1	RN1	2644	-	74.77	0.23	85.30	9.61	228.67
R2	RN1	895	-	25.31	2.38	104.83	96.08	88.62
RN1	<None>	3536	-	70.72	72.40	144.55	-	-
R3	RN1	7	-	0.20	28.07	82.87	93.25	57.35

timepoint 0 h plumbagin treatment

File: X0h_cytoID_at_030.FCS Date: 11-11-2014 Time: 11:57:36 Particles: 20000 Acq.-Time: 249 s partec PAS

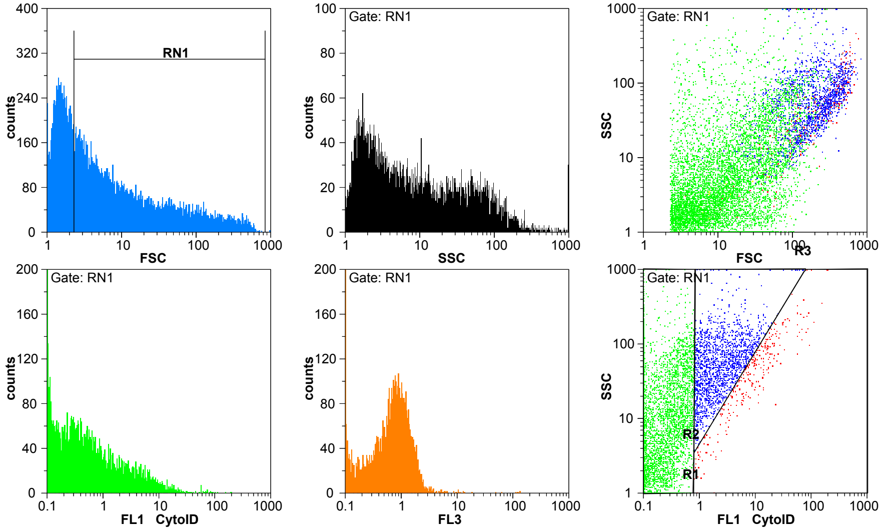

R3: 1.27% R1: 90.80% R2: 7.96%

Region	Gate	Count	Count/ml	%Gated	Mean-x	CV-x%	Mean-y	CV-y%
R1	RN1	11350	-	86.06	0.17	88.70	12.77	401.06
R2	RN1	1592	-	12.07	3.37	135.71	83.47	144.03
RN1	<None>	13188	-	65.94	49.93	197.39	-	-
R3	RN1	252	-	1.91	17.80	152.02	78.89	173.28

timepoint 40 min plumbagin treatment

File: X40 min._008_50f.FCS Date: 03-11-2014 Time: 10:37:35 Particles: 20000 Acq.-Time: 65 s partec PAS

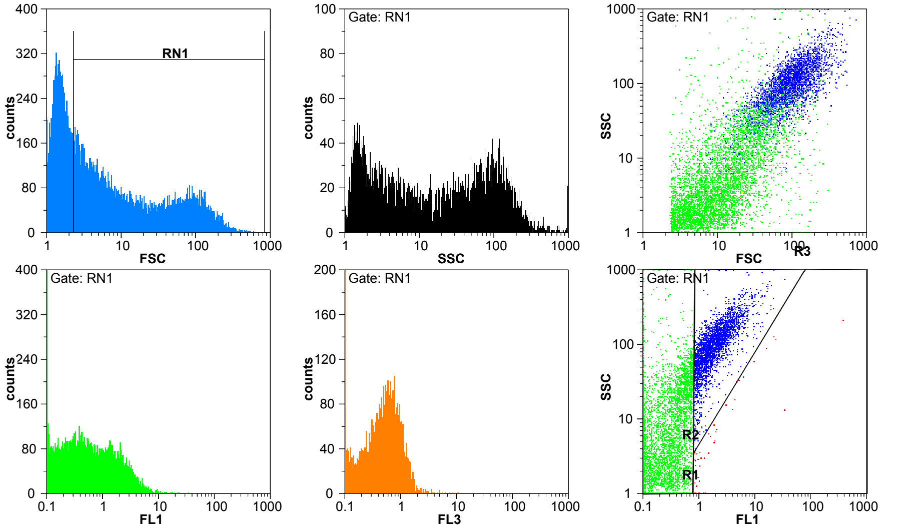

R3: 0.34% R1: 83.52% R2: 16.35%

Region	Gate	Count	Count/ml	%Gated	Mean-x	CV-x%	Mean-y	CV-y%
R1	RN1	9472	-	74.28	0.24	81.16	11.65	460.03
R2	RN1	3269	-	25.64	2.35	87.47	118.46	80.37
RN1	<None>	12752	-	63.76	40.77	154.04	-	-
R3	RN1	49	-	0.38	11.34	477.38	17.28	228.27

timepoint 90 min plumbagin treatment

File: X1.5_Cyto_50f2.FCS Date: 03-11-2014 Time: 11:21:16 Particles: 20000 Acq.-Time: 12 s partec PAS

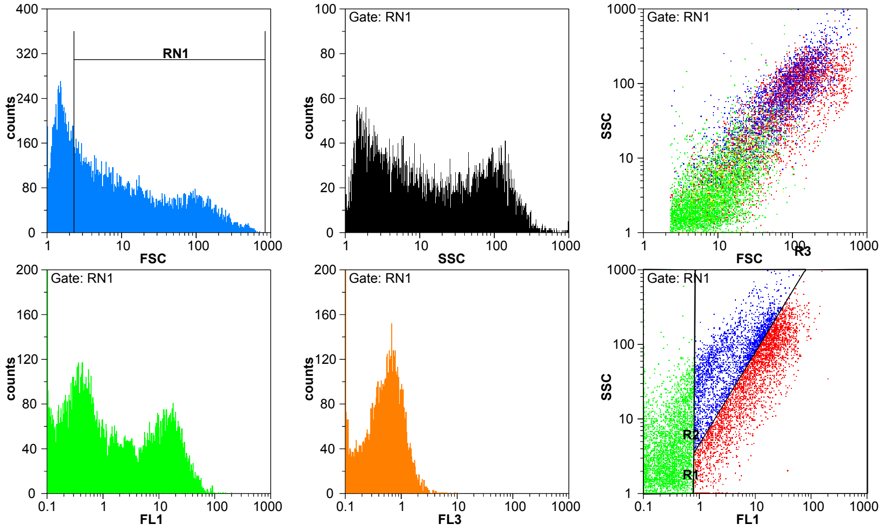

R3: 16.59% R1: 71.09% R2: 12.31%

Region	Gate	Count	Count/ml	%Gated	Mean-x	CV-x%	Mean-y	CV-y%
R1	RN1	8212	-	59.04	0.25	76.91	4.99	409.89
R2	RN1	2447	-	17.59	6.24	101.53	97.73	108.80
RN1	<None>	13909	-	69.55	49.63	170.66	-	-
R3	RN1	3237	-	23.27	15.56	100.71	68.60	107.58

Flow-cytometric analysis - CytoID staining - plumbagin-treated PC3 cells

timepoint 4 h plumbagin treatment

File: X4h_cytoID_50f_2.FCS Date: 03-11-2014 Time: 13:29:57 Particles: 20000 Acq.-Time: 20 s Concentration: 53650 / ml partec PAS

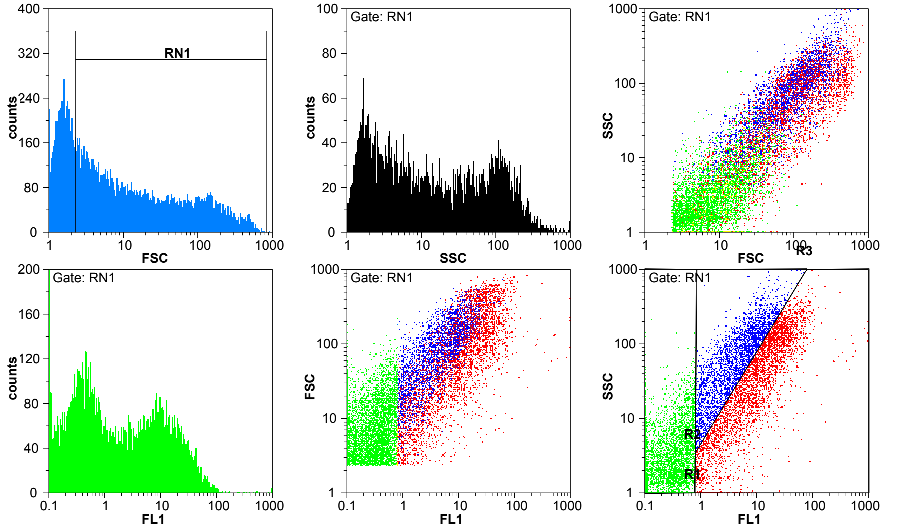

R3: 19.98% **R1: 66.61%** **R2: 13.50%**

Region	Gate	Count	Count/ml	%Gated	Mean-x	CV-x%	Mean-y	CV-y%
R1	RN1	7464	18620	53.38	0.27	74.23	3.61	187.82
R2	RN1	2696	7775	19.28	6.30	100.53	98.74	113.48
RN1	<None>	13984	38055	69.92	61.87	171.25	-	-
R3	RN1	3831	11685	27.40	19.17	234.11	65.22	122.52

timepoint 6 h plumbagin treatment

File: X6h_Cyto_1.FCS Date: 03-11-2014 Time: 15:34:38 Particles: 20000 Acq.-Time: 7 s partec PAS

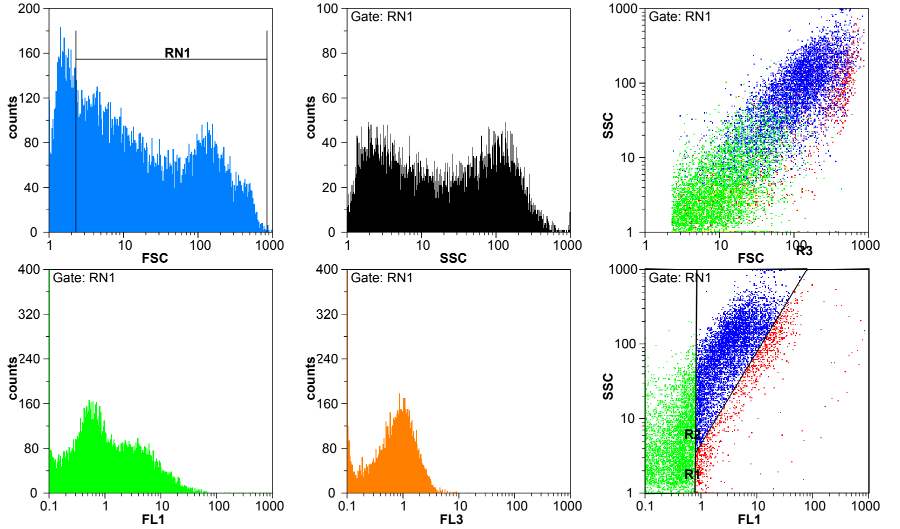

R3: 6.03% **R1: 65.80%** **R2: 28.57%**

Region	Gate	Count	Count/ml	%Gated	Mean-x	CV-x%	Mean-y	CV-y%
R1	RN1	8997	-	57.20	0.31	73.35	5.49	183.31
R2	RN1	5708	-	36.29	4.43	101.74	115.34	96.91
RN1	<None>	15729	-	78.64	78.51	156.21	-	-
R3	RN1	1085	-	6.90	18.58	336.19	61.26	147.29

timepoint 8 h plumbagin treatment

File: X8hod Au_007.FCS Date: 20-05-2014 Time: 16:18:44 Particles: 20000 Acq.-Time: 71 s partec PAS

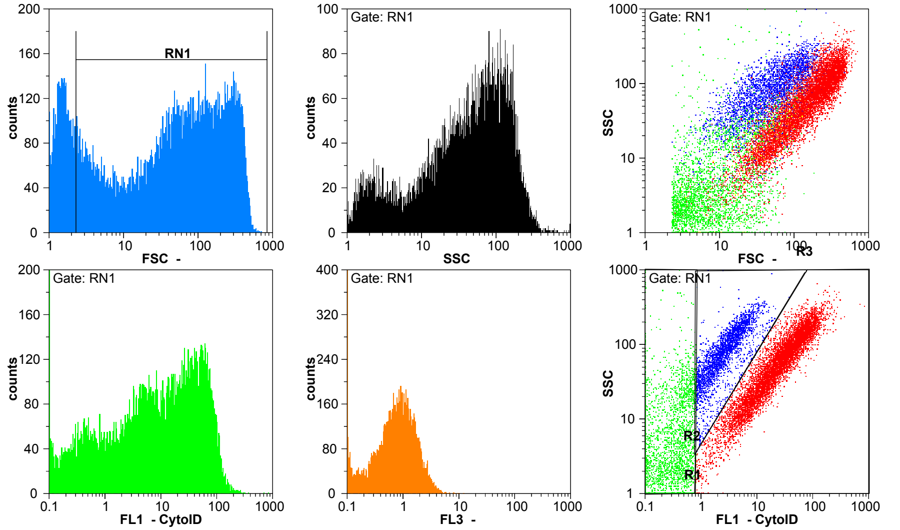

R3: 46.02% **R1: 39.24%** **R2: 14.90%**

Region	Gate	Count	Count/ml	%Gated	Mean-x	CV-x%	Mean-y	CV-y%
R1	RN1	4476	-	26.97	0.27	78.30	10.83	408.57
R2	RN1	2979	-	17.95	3.43	79.99	100.15	75.74
RN1	<None>	16596	-	82.98	115.02	106.36	-	-
R3	RN1	9166	-	55.23	36.72	96.77	77.21	85.82

timepoint 10 h plumbagin treatment

File: X10h_Cyto_004.FCS Date: 03-11-2014 Time: 19:36:39 Particles: 20000 Acq.-Time: 88 s partec PAS

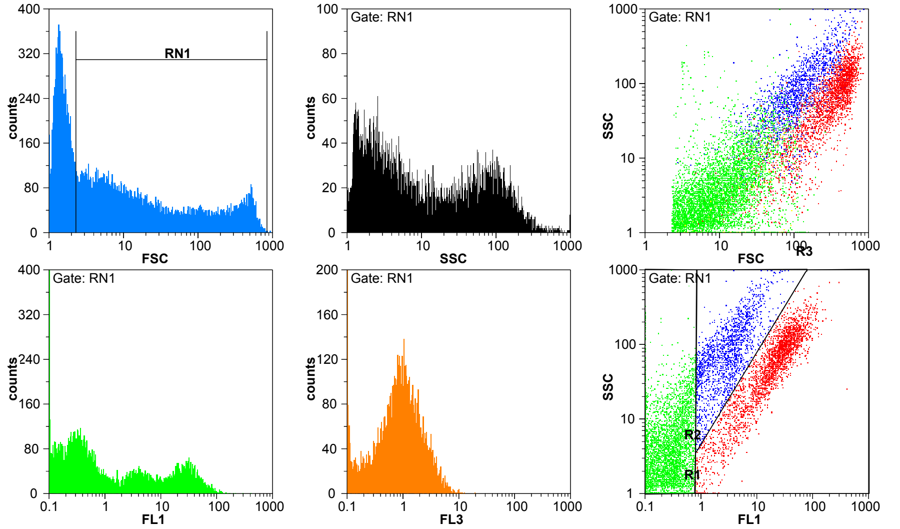

R3: 13.82% **R1: 78.25%** **R2: 8.01%**

Region	Gate	Count	Count/ml	%Gated	Mean-x	CV-x%	Mean-y	CV-y%
R1	RN1	8410	-	66.05	0.23	76.53	5.59	340.10
R2	RN1	1600	-	12.57	4.10	100.26	119.79	118.10
RN1	<None>	12733	-	63.66	97.81	165.60	-	-
R3	RN1	2736	-	21.49	27.06	91.51	76.28	92.08

Flow-cytometric analysis - CytoID staining - plumbagin-treated PC3 cells

timepoint 16 h plumbagin treatment

File: X16h_Cyto_001.FCS Date: 04-11-2014 Time: 10:57:06 Particles: 20000 Acq.-Time: 208 s Concentration: 36965 / ml partec PAS

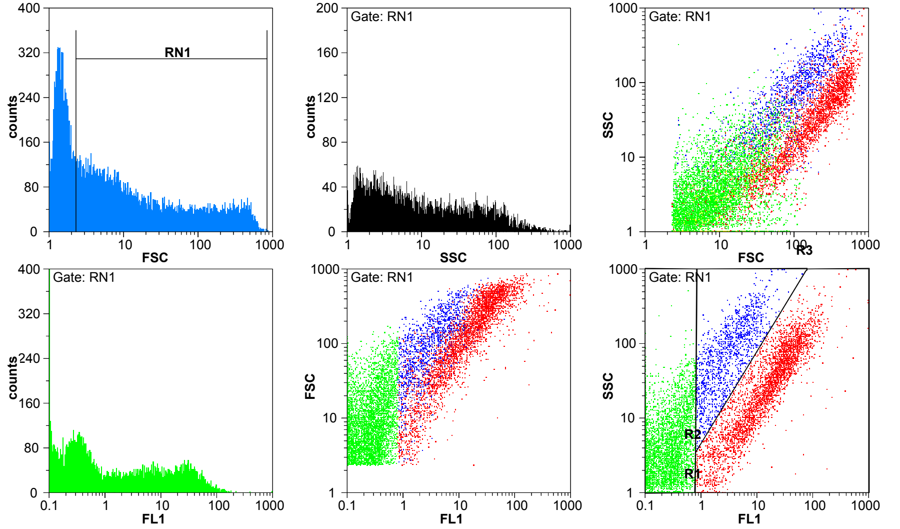

R3: 18.24% R1: 75.41% R2: 6.43%

Region	Gate	Count	Count/ml	%Gated	Mean-x	CV-x%	Mean-y	CV-y%
R1	RN1	8159	16550	62.59	0.22	77.39	4.76	225.12
R2	RN1	1281	2150	9.83	4.40	105.55	113.80	114.86
RN1	<None>	13035	24100	65.17	77.69	172.12	-	-
R3	RN1	3605	5450	27.66	26.59	167.40	48.74	135.18

timepoint 20 h plumbagin treatment

File: X20h_Cyto_002.FCS Date: 04-11-2014 Time: 12:09:22 Particles: 20000 Acq.-Time: 44 s partec PAS

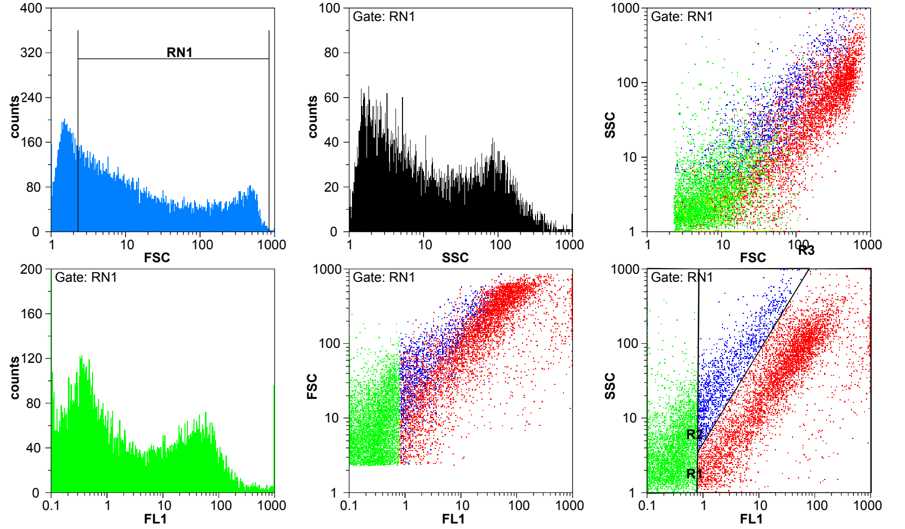

R3: 29.05% R1: 64.33% R2: 6.78%

Region	Gate	Count	Count/ml	%Gated	Mean-x	CV-x%	Mean-y	CV-y%
R1	RN1	8069	-	53.52	0.26	75.58	4.93	421.28
R2	RN1	1345	-	8.92	5.46	140.75	92.29	153.56
RN1	<None>	15076	-	75.38	95.04	166.10	-	-
R3	RN1	5687	-	37.72	70.38	226.21	66.07	134.35

timepoint 24 h plumbagin treatment

File: X24h_Cyto_003.FCS Date: 04-11-2014 Time: 09:45:25 Particles: 20000 Acq.-Time: 94 s Concentration: 74195 / ml partec PAS

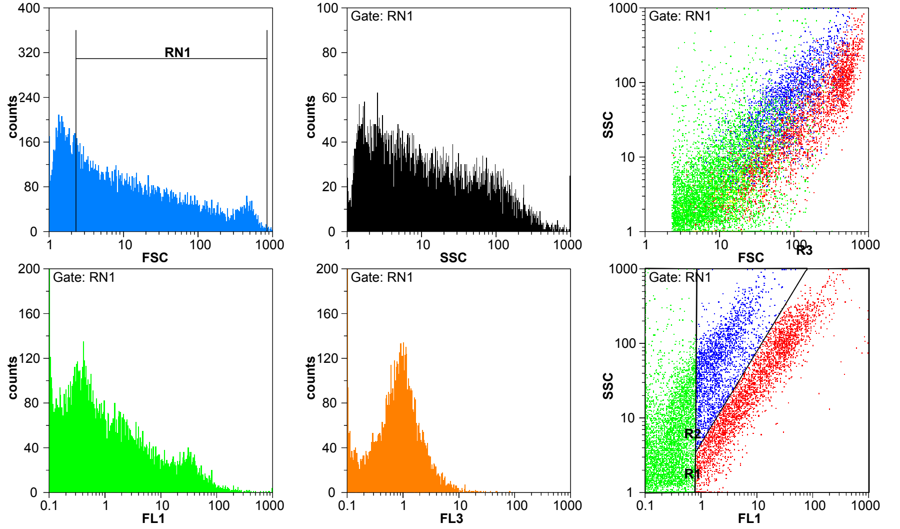

R3: 16.05% R1: 73.60% R2: 10.49%

Region	Gate	Count	Count/ml	%Gated	Mean-x	CV-x%	Mean-y	CV-y%
R1	RN1	9595	34990	64.67	0.24	80.05	11.00	460.82
R2	RN1	2098	7420	14.14	3.10	112.92	96.97	129.20
RN1	<None>	14837	55060	74.19	73.77	185.21	-	-
R3	RN1	3169	12720	21.36	25.39	236.77	67.15	149.29

timepoint 36 h plumbagin treatment

File: X36h_Cyto_001.FCS Date: 04-11-2014 Time: 21:49:39 Particles: 20000 Acq.-Time: 27 s partec PAS

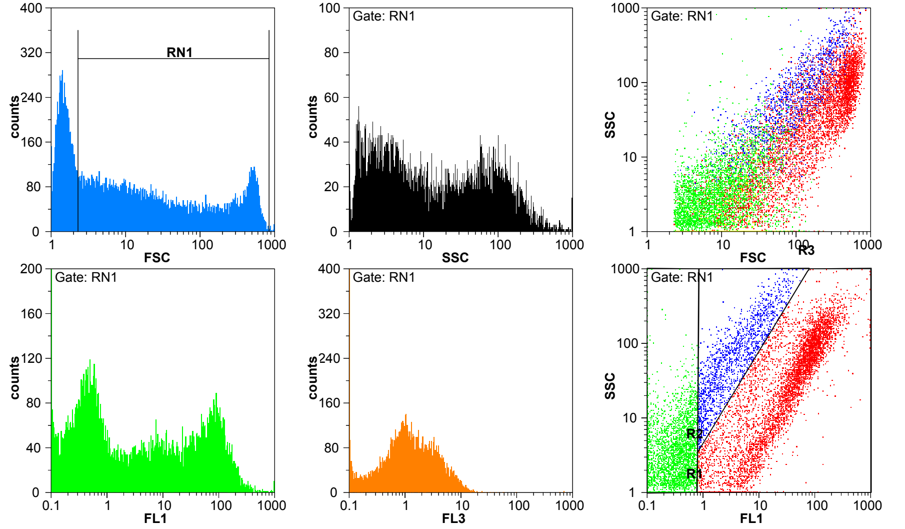

R3: 30.40% R1: 62.78% R2: 7.12%

Region	Gate	Count	Count/ml	%Gated	Mean-x	CV-x%	Mean-y	CV-y%
R1	RN1	6949	-	49.34	0.28	74.51	5.11	611.88
R2	RN1	1420	-	10.08	6.19	120.15	119.46	128.91
RN1	<None>	14085	-	70.42	123.54	149.64	-	-
R3	RN1	5749	-	40.82	61.88	136.41	62.82	137.64

Flow-cytometric analysis - CytoID staining - plumbagin-treated PC3 cells

timepoint 48 h plumbagin treatment

File: X48h_Cyto_001.FCS Date: 05-11-2014 Time: 10:00:12 Particles: 20000 Acq.-Time: 36 s Concentration: 84195 / ml partec PAS

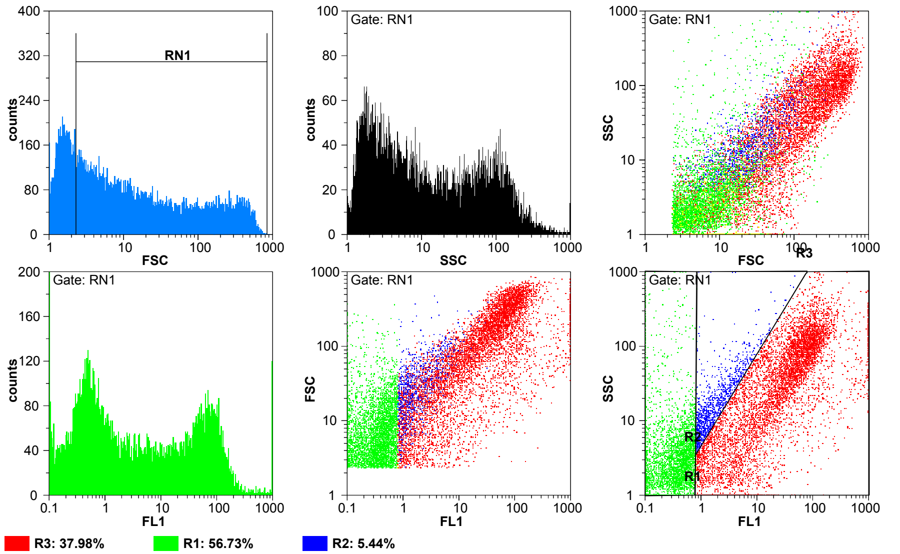

Region	Gate	Count	Count/ml	%Gated	Mean-x	CV-x%	Mean-y	CV-y%
R1	RN1	6684	27695	44.56	0.30	72.29	10.05	570.35
R2	RN1	1080	4585	7.20	2.70	140.16	33.57	244.18
RN1	<None>	15000	63350	75.00	84.84	161.80	-	-
R3	RN1	7253	31130	48.35	68.81	213.89	63.31	139.45

Flow-cytometric analysis - Annexin V/propidium iodide staining - plumbagin-treated PC3 cells

timepoint 0 h plumbagin treatment

File: 0h annex.FCS Date: 15-06-2014 Time: 19:25:44 Particles: 35203 Acq.-Time: 780 s partec PAS

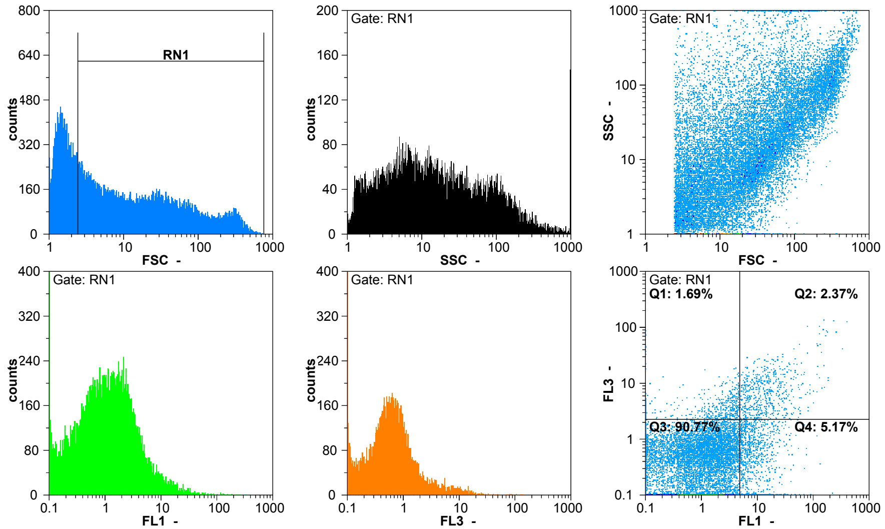

Region	Gate	Count	Count/ml	%Gated	Mean-x	CV-x%	Mean-y	CV-y%
Q1	RN1	403	-	1.69	2.49	60.54	5.40	130.07
Q2	RN1	564	-	2.37	25.21	150.47	10.77	137.50
Q3	RN1	21618	-	90.77	0.99	108.66	0.30	125.10
Q4	RN1	1231	-	5.17	10.73	106.14	0.58	104.59
RN1	<None>	23816	-	67.65	58.82	165.55	-	-

timepoint 20 min plumbagin treatment

File: 20min _annex.FCS Date: 15-06-2014 Time: 17:02:27 Particles: 20184 Acq.-Time: 27 s partec PAS

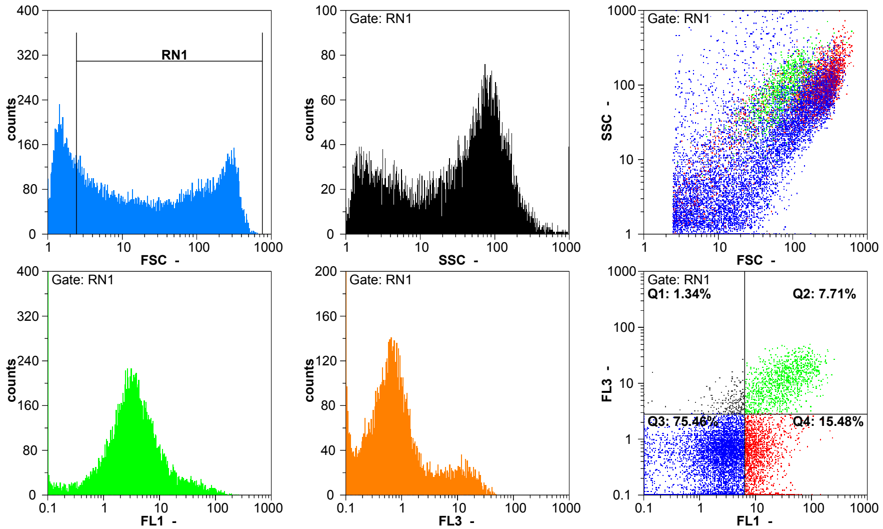

Q4: 12.76% Q2: 5.78% Q3: 80.38%

Region	Gate	Count	Count/ml	%Gated	Mean-x	CV-x%	Mean-y	CV-y%
Q1	RN1	203	-	1.34	3.84	43.48	5.30	55.93
Q2	RN1	1164	-	7.71	41.50	85.45	13.10	64.65
Q3	RN1	11395	-	75.46	2.28	76.93	0.38	122.93
Q4	RN1	2338	-	15.48	13.24	94.39	0.55	109.99
RN1	<None>	15100	-	74.81	103.05	117.51	-	-

timepoint 40 min plumbagin treatment

File: 40min annex.FCS Date: 15-06-2014 Time: 19:10:18 Particles: 20546 Acq.-Time: 32 s Concentration: 110 / ml partec PAS

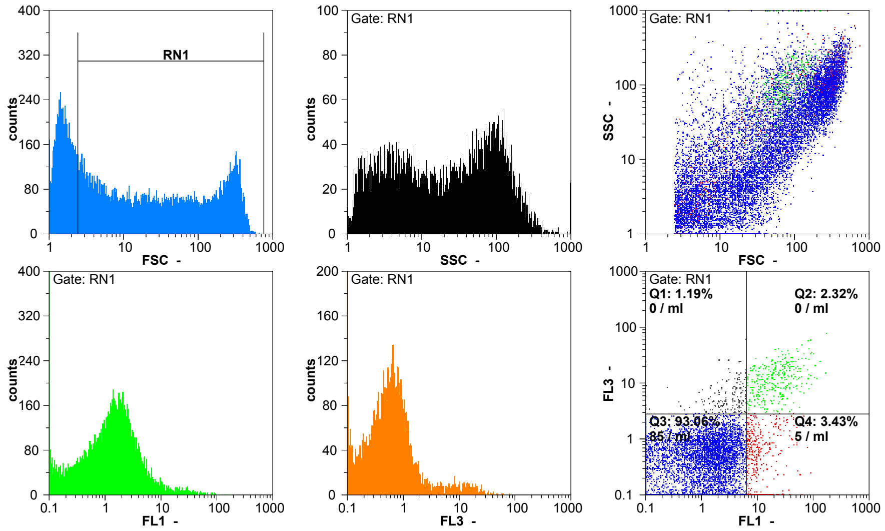

Q4: 3.02% Q2: 1.65% Q3: 94.46%

Region	Gate	Count	Count/ml	%Gated	Mean-x	CV-x%	Mean-y	CV-y%
Q1	RN1	173	0	1.19	3.42	53.12	7.31	88.18
Q2	RN1	338	0	2.32	28.53	84.75	13.98	68.55
Q3	RN1	13529	85	93.06	1.26	104.76	0.31	125.06
Q4	RN1	498	5	3.43	13.71	85.43	0.63	110.23
RN1	<None>	14538	90	70.76	95.85	127.18	-	-

timepoint 90 min plumbagin treatment

File: 1hod annex.FCS Date: 20-05-2014 Time: 10:27:15 Particles: 21410 Acq.-Time: 135 s partec PAS

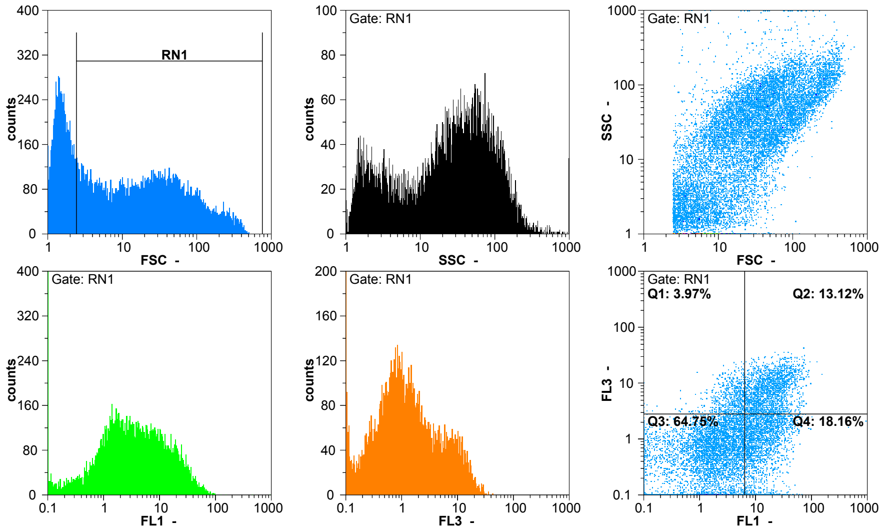

Region	Gate	Count	Count/ml	%Gated	Mean-x	CV-x%	Mean-y	CV-y%
Q1	RN1	588	-	3.97	3.84	41.63	6.43	56.89
Q2	RN1	1944	-	13.12	22.46	68.25	9.11	61.57
Q3	RN1	9592	-	64.75	1.87	88.70	0.46	122.00
Q4	RN1	2691	-	18.16	16.30	138.40	1.01	82.69
RN1	<None>	14815	-	69.20	52.47	144.79	-	-

Flow-cytometric analysis - Annexin V/propidium iodide staining - plumbagin-treated PC3 cells

timepoint 0 h plumbagin treatment

File: 2hod annex.FCS Date: 20-05-2014 Time: 11:26:06 Particles: 30237 Acq.-Time: 327 s Concentration: 9015 / ml partec PAS

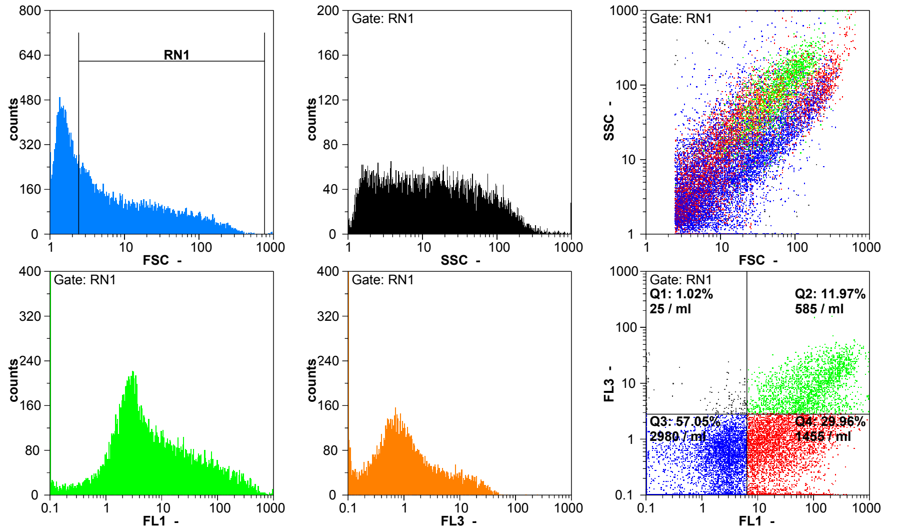

Q4: 27.16% Q2: 7.31% Q3: 64.64%

Region	Gate	Count	Count/ml	%Gated	Mean-x	CV-x%	Mean-y	CV-y%
Q1	RN1	187	25	1.02	1.87	113.77	8.17	86.79
Q2	RN1	2202	585	11.97	152.01	104.01	11.80	96.58
Q3	RN1	10492	2980	57.05	2.28	73.66	0.33	130.29
Q4	RN1	5511	1455	29.96	41.12	149.27	0.75	99.96
RN1	<None>	18392	5045	60.83	37.61	167.02	-	-

timepoint 20 min plumbagin treatment

File: 3hod annex.FCS Date: 20-05-2014 Time: 12:06:02 Particles: 20000 Acq.-Time: 47 s partec PAS

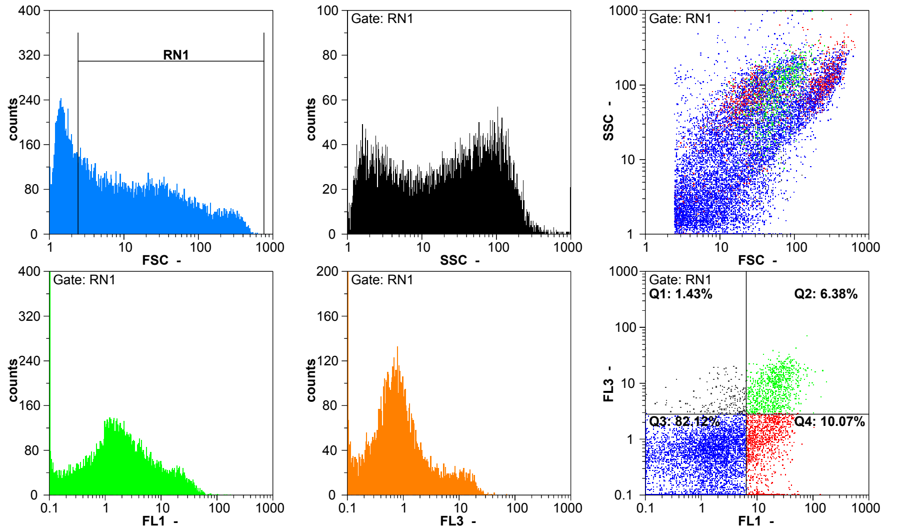

Q4: 7.54% Q2: 4.52% Q3: 86.91%

Region	Gate	Count	Count/ml	%Gated	Mean-x	CV-x%	Mean-y	CV-y%
Q1	RN1	202	-	1.43	3.01	61.55	6.38	76.55
Q2	RN1	904	-	6.38	23.93	69.23	9.98	64.02
Q3	RN1	11641	-	82.12	1.35	110.92	0.35	125.71
Q4	RN1	1428	-	10.07	15.02	65.42	0.94	87.78
RN1	<None>	14175	-	70.88	52.79	162.07	-	-

timepoint 40 min plumbagin treatment

File: 4hod annex.FCS Date: 20-05-2014 Time: 12:54:19 Particles: 20000 Acq.-Time: 56 s partec PAS

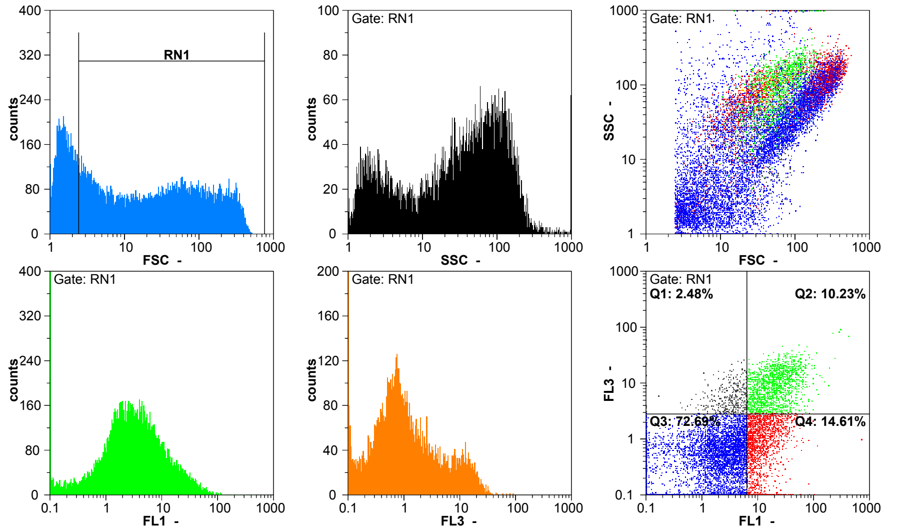

Q4: 11.36% Q2: 7.56% Q3: 79.22%

Region	Gate	Count	Count/ml	%Gated	Mean-x	CV-x%	Mean-y	CV-y%
Q1	RN1	366	-	2.48	3.64	45.45	6.74	77.69
Q2	RN1	1511	-	10.23	25.70	97.27	10.91	70.64
Q3	RN1	10740	-	72.69	2.06	83.77	0.36	132.58
Q4	RN1	2158	-	14.61	13.72	132.69	0.88	94.01
RN1	<None>	14775	-	73.88	77.39	128.32	-	-

timepoint 90 min plumbagin treatment

File: 5hod annex.FCS Date: 20-05-2014 Time: 13:53:59 Particles: 20000 Acq.-Time: 62 s partec PAS

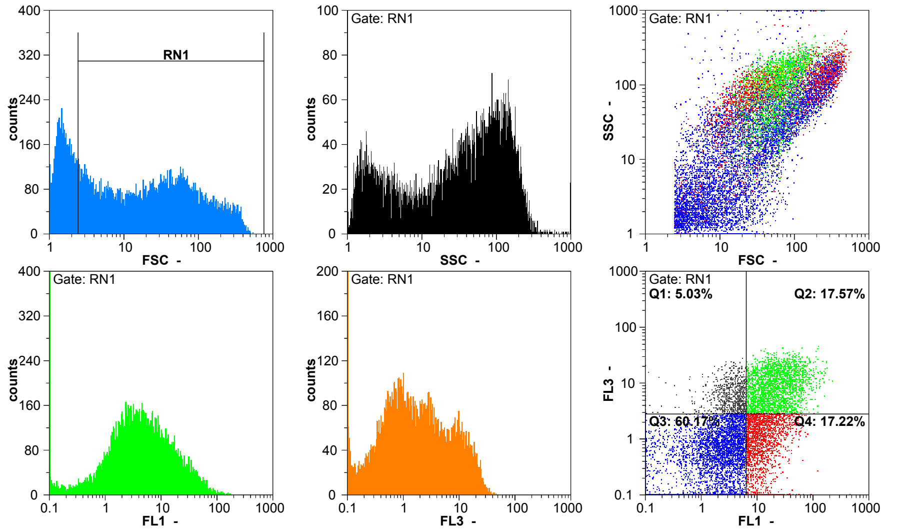

Q4: 13.51% Q2: 12.96% Q3: 69.77%

Region	Gate	Count	Count/ml	%Gated	Mean-x	CV-x%	Mean-y	CV-y%
Q1	RN1	742	-	5.03	3.76	43.02	7.08	59.31
Q2	RN1	2591	-	17.57	27.96	85.31	10.39	65.98
Q3	RN1	8874	-	60.17	2.15	80.85	0.46	129.74
Q4	RN1	2540	-	17.22	14.58	94.33	1.03	86.29
RN1	<None>	14747	-	73.73	63.52	136.60	-	-

Flow-cytometric analysis - Annexin V/propidium iodide staining - plumbagin-treated PC3 cells

timepoint 6 h plumbagin treatment

File: 6hod annex.FCS Date: 20-05-2014 Time: 14:58:54 Particles: 20000 Acq.-Time: 43 s partec PAS

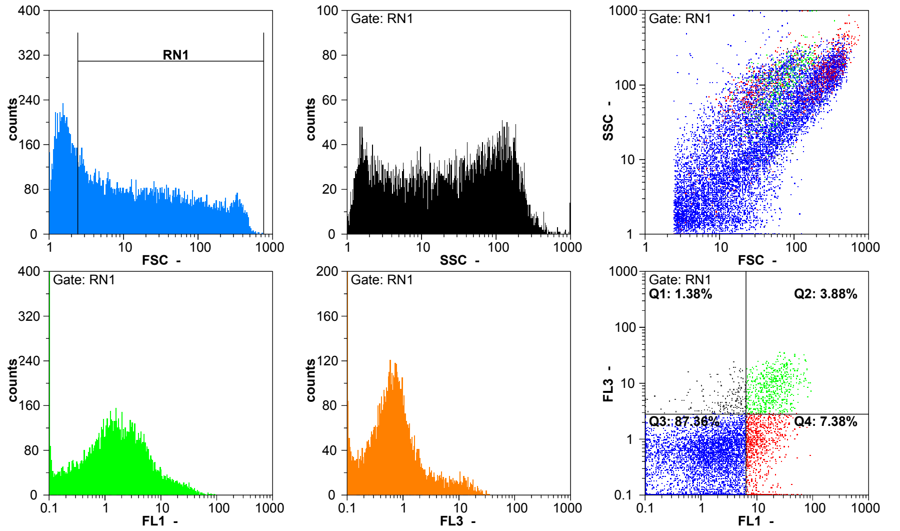

Q4: 5.44% Q2: 2.77% Q3: 90.79%

Region	Gate	Count	Count/ml	%Gated	Mean-x	CV-x%	Mean-y	CV-y%
Q1	RN1	196	-	1.38	3.20	58.43	6.30	62.40
Q2	RN1	553	-	3.88	22.95	65.66	10.07	63.43
Q3	RN1	12435	-	87.36	1.41	109.04	0.32	127.92
Q4	RN1	1051	-	7.38	12.98	65.76	0.85	93.27
RN1	<None>	14235	-	71.17	71.14	152.19	-	-

timepoint 7 h plumbagin treatment

File: 7hod annex.FCS Date: 20-05-2014 Time: 16:00:33 Particles: 20000 Acq.-Time: 56 s partec PAS

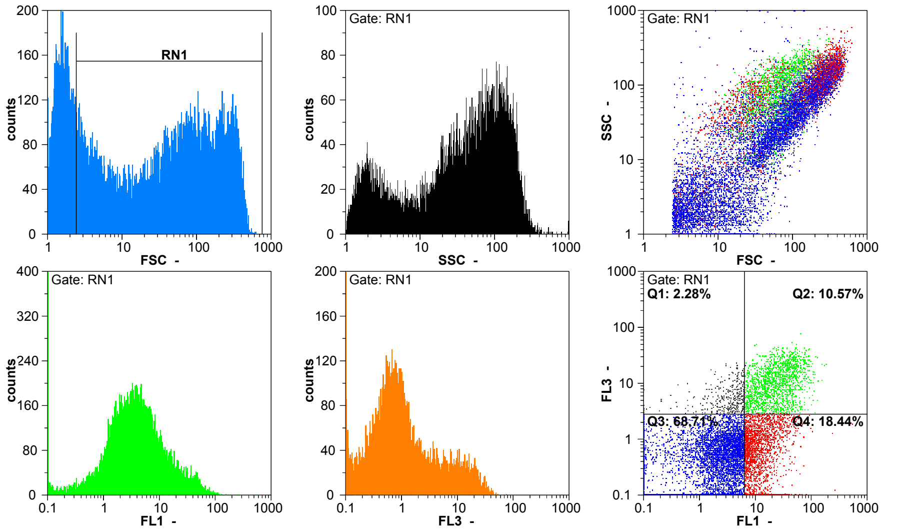

Q4: 14.99% Q2: 8.09% Q3: 75.13%

Region	Gate	Count	Count/ml	%Gated	Mean-x	CV-x%	Mean-y	CV-y%
Q1	RN1	349	-	2.28	3.75	47.26	6.45	60.40
Q2	RN1	1618	-	10.57	29.77	74.21	12.40	70.71
Q3	RN1	10515	-	68.71	2.39	72.91	0.36	126.84
Q4	RN1	2822	-	18.44	14.08	109.85	0.72	103.42
RN1	<None>	15304	-	76.52	101.83	112.62	-	-

timepoint 9 h plumbagin treatment

File: 9hod annex.FCS Date: 20-05-2014 Time: 17:52:19 Particles: 20000 Acq.-Time: 36 s partec PAS

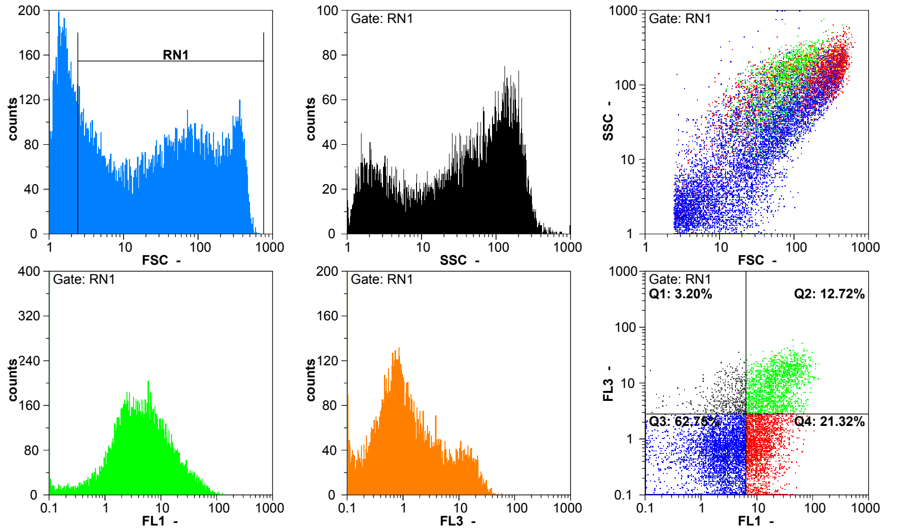

Q4: 16.60% Q2: 9.55% Q3: 71.40%

Region	Gate	Count	Count/ml	%Gated	Mean-x	CV-x%	Mean-y	CV-y%
Q1	RN1	481	-	3.20	3.54	51.59	6.78	64.41
Q2	RN1	1910	-	12.72	27.26	71.71	11.39	67.74
Q3	RN1	9419	-	62.75	2.34	76.63	0.42	127.34
Q4	RN1	3201	-	21.32	13.29	62.90	0.80	96.81
RN1	<None>	15011	-	75.06	99.30	123.87	-	-

timepoint 11 h plumbagin treatment

File: 11hod annex.FCS Date: 20-05-2014 Time: 19:57:30 Particles: 20000 Acq.-Time: 37 s partec PAS

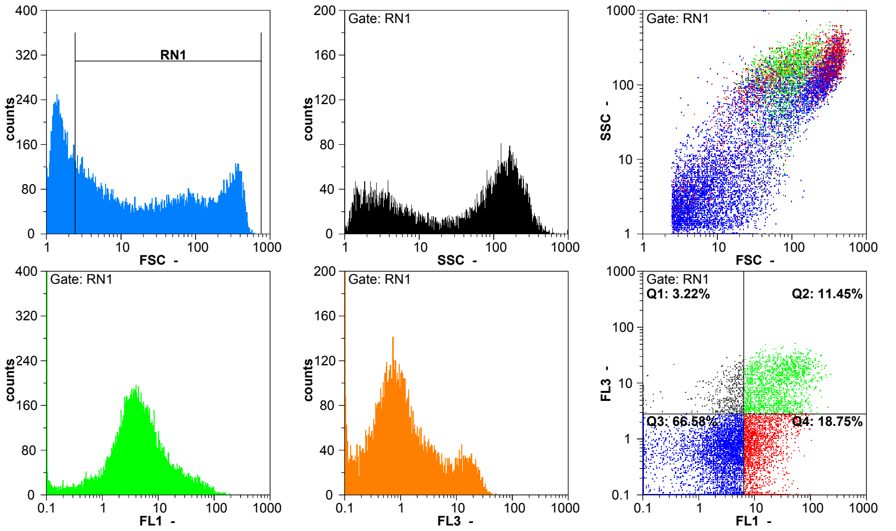

Q4: 14.18% Q2: 8.04% Q3: 75.44%

Region	Gate	Count	Count/ml	%Gated	Mean-x	CV-x%	Mean-y	CV-y%
Q1	RN1	453	-	3.22	3.64	50.27	7.54	66.76
Q2	RN1	1608	-	11.45	34.92	87.03	12.11	67.72
Q3	RN1	9352	-	66.58	2.39	77.48	0.43	120.65
Q4	RN1	2634	-	18.75	14.51	81.10	0.82	95.59
RN1	<None>	14047	-	70.23	103.84	128.27	-	-

Flow-cytometric analysis - Annexin V/propidium iodide staining - plumbagin-treated PC3 cells

timepoint 13 h plumbagin treatment

File: 13hod annex.FCS Date: 20-05-2014 Time: 21:47:41 Particles: 20000 Acq.-Time: 21 s Concentration: 58025 / ml partec PAS

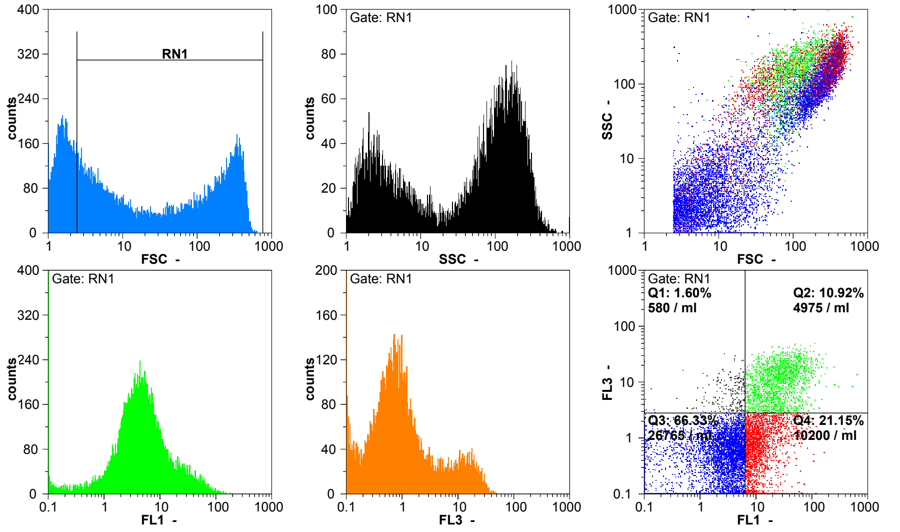

Q4: 16.63% Q2: 7.97% Q3: 74.19%

Region	Gate	Count	Count/ml	%Gated	Mean-x	CV-x%	Mean-y	CV-y%
Q1	RN1	233	580	1.60	3.77	47.86	7.40	67.11
Q2	RN1	1592	4975	10.92	34.18	99.07	12.66	64.88
Q3	RN1	9672	26765	66.33	2.65	71.07	0.39	118.65
Q4	RN1	3084	10200	21.15	13.89	99.36	0.74	97.73
RN1	<None>	14581	42520	72.91	125.68	113.59	-	-

timepoint 28 h plumbagin treatment

File: 28hod annex.FCS Date: 21-05-2014 Time: 13:12:47 Particles: 20000 Acq.-Time: 16 s partec PAS

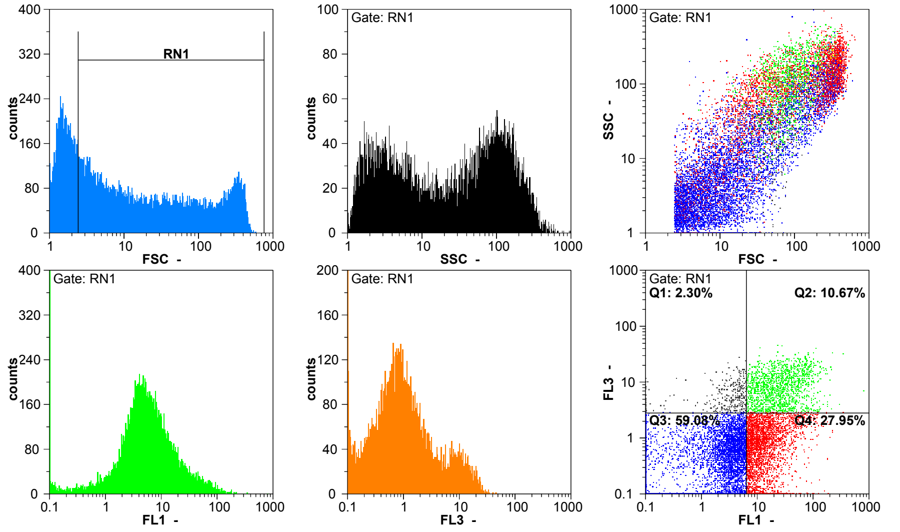

Q4: 21.89% Q2: 7.56% Q3: 68.89%

Region	Gate	Count	Count/ml	%Gated	Mean-x	CV-x%	Mean-y	CV-y%
Q1	RN1	326	-	2.30	3.63	50.36	6.83	58.26
Q2	RN1	1511	-	10.67	36.23	107.65	9.85	62.42
Q3	RN1	8369	-	59.08	2.72	71.44	0.44	120.92
Q4	RN1	3960	-	27.95	16.87	115.90	0.79	96.13
RN1	<None>	14166	-	70.83	86.27	140.14	-	-

timepoint 48 h plumbagin treatment

File: 48hod annex.FCS Date: 22-05-2014 Time: 13:37:54 Particles: 20000 Acq.-Time: 20 s partec PAS

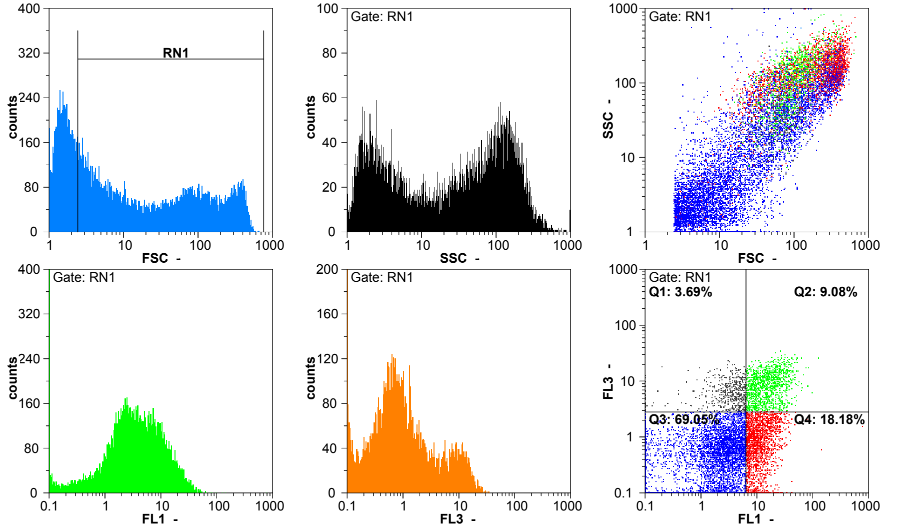

Q4: 12.90% Q2: 6.24% Q3: 78.30%

Region	Gate	Count	Count/ml	%Gated	Mean-x	CV-x%	Mean-y	CV-y%
Q1	RN1	507	-	3.69	3.55	50.52	7.05	53.06
Q2	RN1	1247	-	9.08	18.18	61.45	9.63	55.30
Q3	RN1	9482	-	69.05	2.07	84.39	0.38	128.12
Q4	RN1	2497	-	18.18	12.31	136.98	0.82	90.13
RN1	<None>	13733	-	68.67	89.54	137.42	-	-

Flow-cytometric analysis - Syto16 staining - plumbagin-treated PC3 cells

timepoint 0 h plumbagin treatment

File: 0h1.FCS   Date: 11-11-2014   Time: 12:05:45   Particles: 20000   Acq.-Time: 23 s   partec PAS

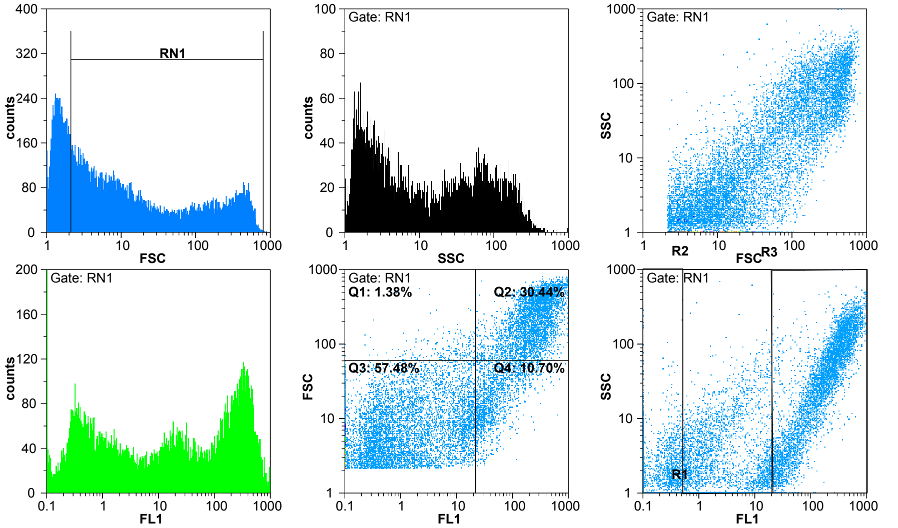

Region	Gate	Count	Count/ml	%Gated	Mean-x	CV-x%	Mean-y	CV-y%
Q1	RN1	201	-	1.38	8.99	73.56	102.54	62.69
Q2	RN1	4421	-	30.44	311.31	60.24	282.83	58.43
Q3	RN1	8350	-	57.48	2.92	173.88	9.40	105.50
Q4	RN1	1554	-	10.70	73.66	107.08	25.24	63.84
R1	RN1	4186	-	28.82	0.20	64.03	2.63	426.06
R2	RN1	4302	-	29.62	5.57	105.23	5.16	364.97
R3	RN1	6047	-	41.63	246.78	79.68	70.87	110.69
RN1	<None>	14526	-	72.63	95.60	161.79	-	-

timepoint 40 min plumbagin treatment

File: N40min_Syto_30f3.FCS   Date: 03-11-2014   Time: 10:59:46   Particles: 20000   Acq.-Time: 40 s   Concentration: 79945 / ml   partec PAS

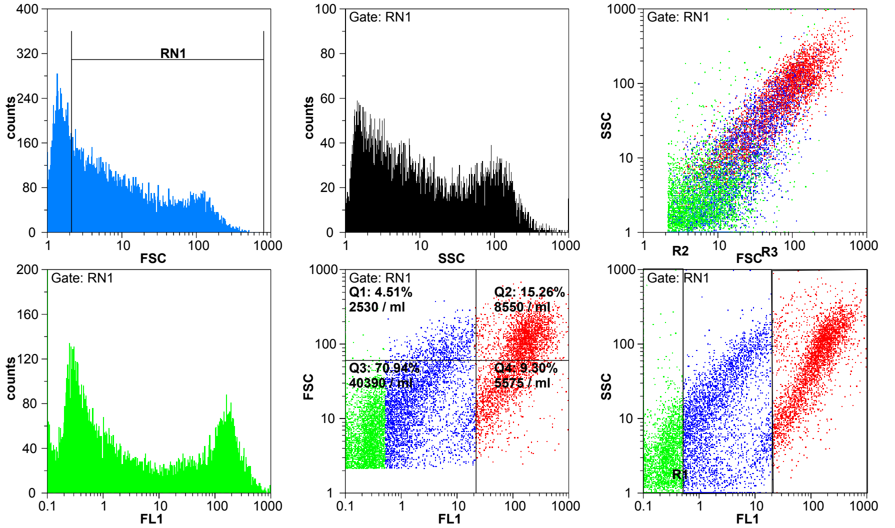

R3: 17.79%   R1: 62.63%   R2: 19.56%

Region	Gate	Count	Count/ml	%Gated	Mean-x	CV-x%	Mean-y	CV-y%
Q1	RN1	646	2530	4.51	6.89	81.57	105.75	44.42
Q2	RN1	2188	8550	15.26	208.17	64.18	145.09	53.28
Q3	RN1	10171	40390	70.94	1.20	249.37	10.92	105.92
Q4	RN1	1333	5575	9.30	93.83	105.05	29.01	55.96
R1	RN1	7124	27585	49.69	0.19	63.06	6.34	729.19
R2	RN1	3660	15205	25.53	3.97	119.74	23.62	191.88
R3	RN1	3548	14225	24.75	163.80	81.60	96.71	95.27
RN1	<None>	14338	57045	71.69	37.35	160.98	-	-

timepoint 90 min plumbagin treatment

File: NNN1.5h_Syto50f4.FCS   Date: 03-11-2014   Time: 11:16:34   Particles: 20000   Acq.-Time: 46 s   partec PAS

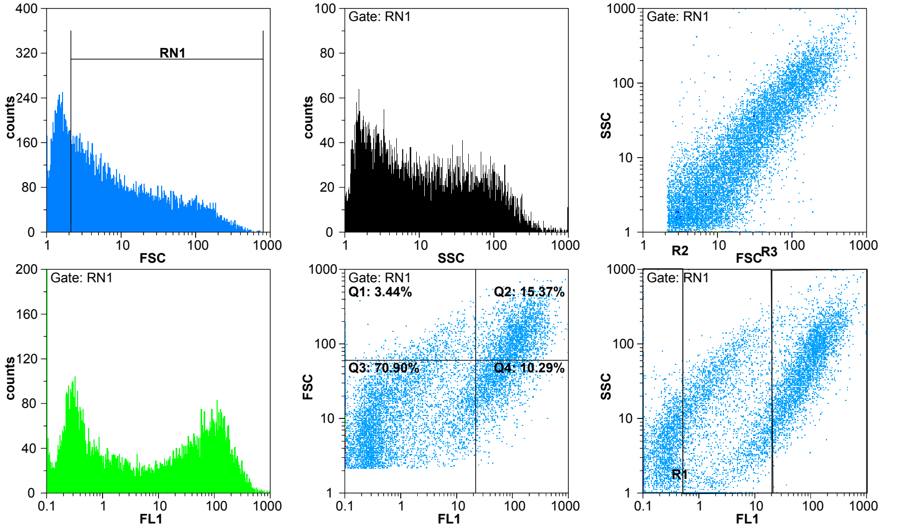

Region	Gate	Count	Count/ml	%Gated	Mean-x	CV-x%	Mean-y	CV-y%
Q1	RN1	502	-	3.44	5.83	97.23	107.10	50.12
Q2	RN1	2246	-	15.37	156.52	71.46	162.13	61.84
Q3	RN1	10358	-	70.90	1.42	260.58	10.39	106.21
Q4	RN1	1504	-	10.29	68.12	84.48	32.80	44.42
R1	RN1	7874	-	53.89	0.16	65.79	5.52	698.39
R2	RN1	2933	-	20.08	5.19	108.05	24.15	175.98
R3	RN1	3796	-	25.98	119.86	86.24	87.49	110.78
RN1	<None>	14610	-	73.05	39.35	176.56	-	-

timepoint 4 h plumbagin treatment

File: NNN4h_Syto50fsuper.FCS   Date: 03-11-2014   Time: 13:22:50   Particles: 20000   Acq.-Time: 15 s   Concentration: 61405 / ml   partec PAS

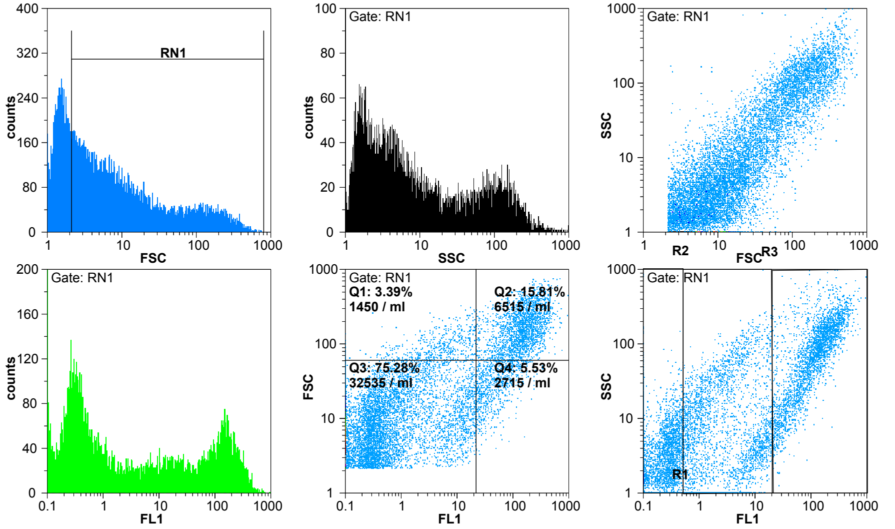

Region	Gate	Count	Count/ml	%Gated	Mean-x	CV-x%	Mean-y	CV-y%
Q1	RN1	483	1450	3.39	6.87	84.08	104.25	43.54
Q2	RN1	2251	6515	15.81	179.59	62.94	197.72	59.55
Q3	RN1	10721	32535	75.28	1.35	258.49	9.85	104.39
Q4	RN1	787	2715	5.53	61.50	73.29	31.76	48.44
R1	RN1	8080	23845	56.73	0.18	65.40	3.38	435.93
R2	RN1	3085	10005	21.66	5.03	108.64	18.82	191.15
R3	RN1	3073	9355	21.58	147.55	76.40	110.89	98.96
RN1	<None>	14242	43215	71.21	43.96	191.66	-	-

Flow-cytometric analysis - Syto16 staining - plumbagin-treated PC3 cells

timepoint 6 h plumbagin treatment

File: NNN6h_syto_50f3_hezke.FCS Date: 03-11-2014 Time: 15:27:31 Particles: 20000 Acq.-Time: 8 s partec PAS

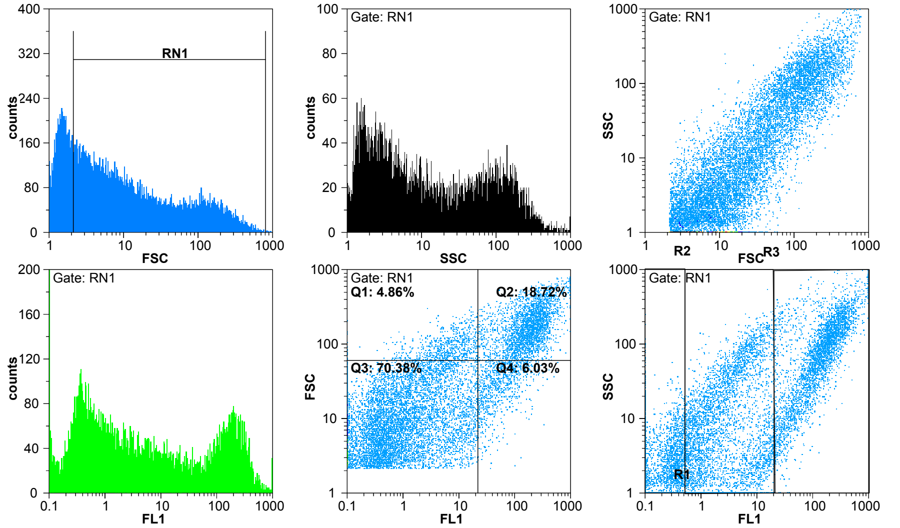

Region	Gate	Count	Count/ml	%Gated	Mean-x	CV-x%	Mean-y	CV-y%
Q1	RN1	735	-	4.86	7.95	70.40	101.11	40.82
Q2	RN1	2830	-	18.72	226.77	72.80	208.56	62.60
Q3	RN1	10639	-	70.38	1.73	207.92	11.17	104.78
Q4	RN1	912	-	6.03	71.67	84.65	31.40	49.77
R1	RN1	6380	-	42.21	0.19	68.62	2.87	217.08
R2	RN1	4940	-	32.68	4.44	109.45	19.51	177.02
R3	RN1	3792	-	25.09	186.77	86.27	123.45	105.42
RN1	<None>	15116	-	75.58	53.72	179.39	-	-

timepoint 8 h plumbagin treatment

File: NNN8h_syto_2.FCS Date: 03-11-2014 Time: 17:09:31 Particles: 20000 Acq.-Time: 38 s partec PAS

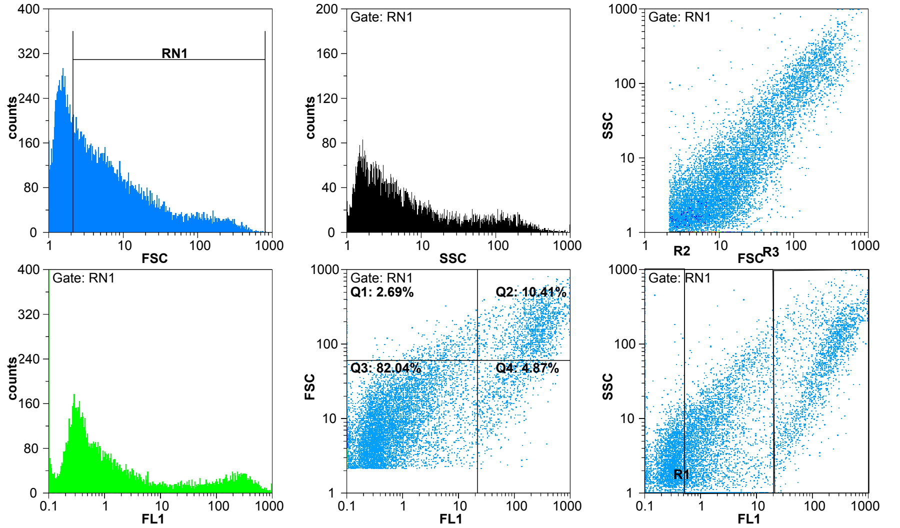

Region	Gate	Count	Count/ml	%Gated	Mean-x	CV-x%	Mean-y	CV-y%
Q1	RN1	369	-	2.69	7.23	82.01	101.11	43.97
Q2	RN1	1430	-	10.41	286.47	69.84	200.66	60.82
Q3	RN1	11273	-	82.04	1.03	232.79	9.89	104.34
Q4	RN1	669	-	4.87	94.78	87.94	28.02	56.89
R1	RN1	7572	-	55.11	0.21	59.96	4.34	662.95
R2	RN1	4048	-	29.46	3.01	129.59	12.40	194.26
R3	RN1	2118	-	15.41	223.55	86.62	117.39	108.99
RN1	<None>	13741	-	68.70	33.08	217.83	-	-

timepoint 10 h plumbagin treatment

File: NNN10h_syto_002.FCS Date: 03-11-2014 Time: 19:19:49 Particles: 20000 Acq.-Time: 21 s partec PAS

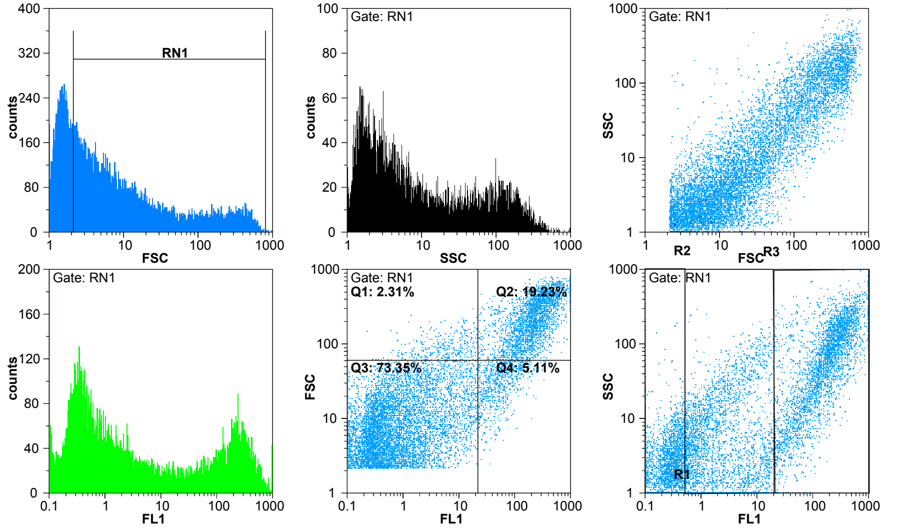

Region	Gate	Count	Count/ml	%Gated	Mean-x	CV-x%	Mean-y	CV-y%
Q1	RN1	326	-	2.31	7.24	79.73	97.87	37.89
Q2	RN1	2711	-	19.23	275.90	69.71	273.72	59.26
Q3	RN1	10341	-	73.35	1.38	224.54	9.63	104.92
Q4	RN1	721	-	5.11	67.74	77.06	32.37	47.49
R1	RN1	6670	-	47.31	0.19	64.22	3.14	537.59
R2	RN1	3972	-	28.17	3.71	120.43	9.92	210.50
R3	RN1	3455	-	24.51	230.77	83.39	99.74	104.39
RN1	<None>	14099	-	70.50	63.62	198.10	-	-

timepoint 16 h h plumbagin treatment

File: NNN16h_syto_004.FCS Date: 04-11-2014 Time: 10:36:02 Particles: 20000 Acq.-Time: 64 s partec PAS

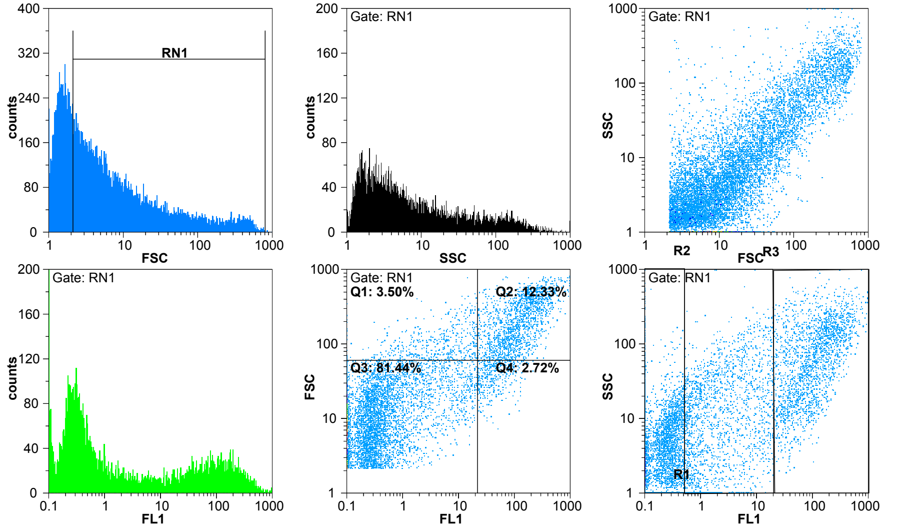

Region	Gate	Count	Count/ml	%Gated	Mean-x	CV-x%	Mean-y	CV-y%
Q1	RN1	475	-	3.50	6.52	95.45	120.79	64.05
Q2	RN1	1675	-	12.33	176.84	85.21	265.42	64.18
Q3	RN1	11061	-	81.44	0.65	336.02	10.08	107.52
Q4	RN1	370	-	2.72	52.80	61.20	38.44	35.37
R1	RN1	9569	-	70.46	0.15	65.88	5.63	519.45
R2	RN1	1953	-	14.38	4.37	118.49	26.62	209.93
R3	RN1	2057	-	15.15	153.62	94.43	108.34	107.06
RN1	<None>	13581	-	67.91	46.21	227.71	-	-

Flow-cytometric analysis - Syto16 staining - plumbagin-treated PC3 cells

timepoint 20 h plumbagin treatment

File: NNN20h_syto_005.FCS   Date: 04-11-2014   Time: 11:52:21   Particles: 20000   Acq.-Time: 16 s   partec PAS

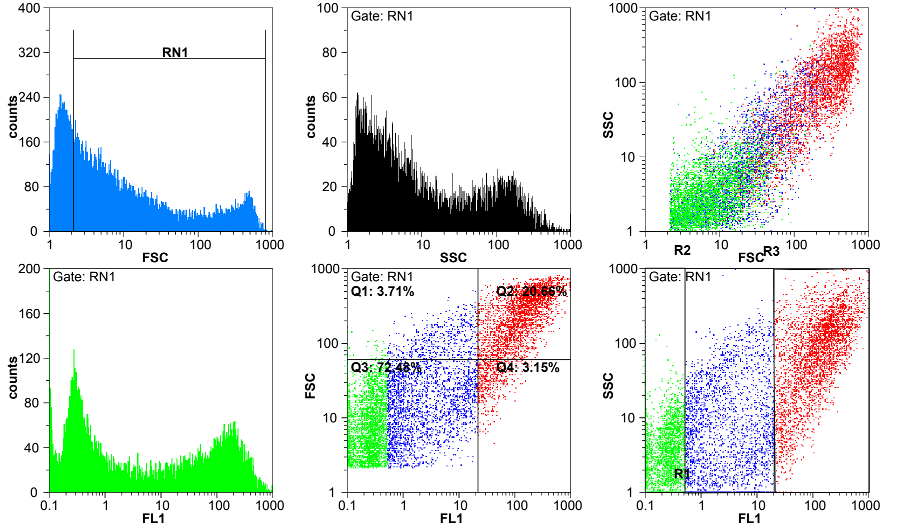

R3: 17.51%   **R1: 67.59%**   **R2: 14.89%**

Region	Gate	Count	Count/ml	%Gated	Mean-x	CV-x%	Mean-y	CV-y%
Q1	RN1	539	-	3.71	6.59	93.43	122.94	53.84
Q2	RN1	2999	-	20.66	191.15	78.23	314.73	54.78
Q3	RN1	10524	-	72.48	1.07	284.88	10.44	103.62
Q4	RN1	457	-	3.15	46.77	60.66	35.55	40.08
R1	RN1	8399	-	57.85	0.17	66.06	3.20	218.83
R2	RN1	2627	-	18.09	4.80	111.43	19.46	237.55
R3	RN1	3491	-	24.04	170.53	86.80	125.83	102.72
RN1	<None>	14519	-	72.59	78.26	186.97	-	-

timepoint 24 h plumbagin treatment

File: NNN24h_syto_1.FCS   Date: 04-11-2014   Time: 09:21:32   Particles: 20000   Acq.-Time: 10 s   partec PAS

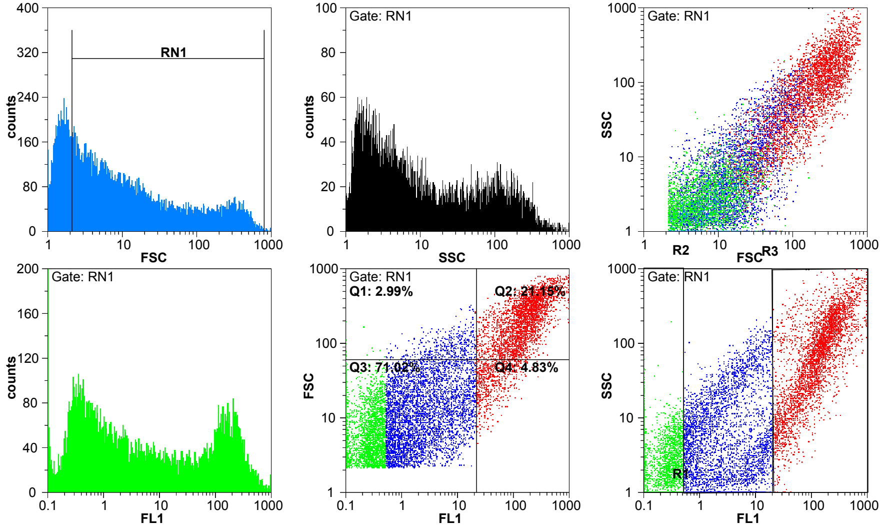

R3: 19.80%   **R1: 53.49%**   **R2: 26.70%**

Region	Gate	Count	Count/ml	%Gated	Mean-x	CV-x%	Mean-y	CV-y%
Q1	RN1	450	-	2.99	8.62	69.26	98.91	41.23
Q2	RN1	3183	-	21.15	209.88	75.22	262.24	59.96
Q3	RN1	10687	-	71.02	1.72	205.56	10.65	102.36
Q4	RN1	727	-	4.83	53.89	71.15	35.46	40.71
R1	RN1	6396	-	42.51	0.19	67.41	2.51	154.30
R2	RN1	4713	-	31.32	4.34	112.47	11.26	190.22
R3	RN1	3935	-	26.15	179.83	86.61	123.69	106.99
RN1	<None>	15047	-	75.23	67.71	185.46	-	-

timepoint 36 h plumbagin treatment

File: NNN36h_syto_002.FCS   Date: 04-11-2014   Time: 21:33:44   Particles: 20000   Acq.-Time: 22 s   partec PAS

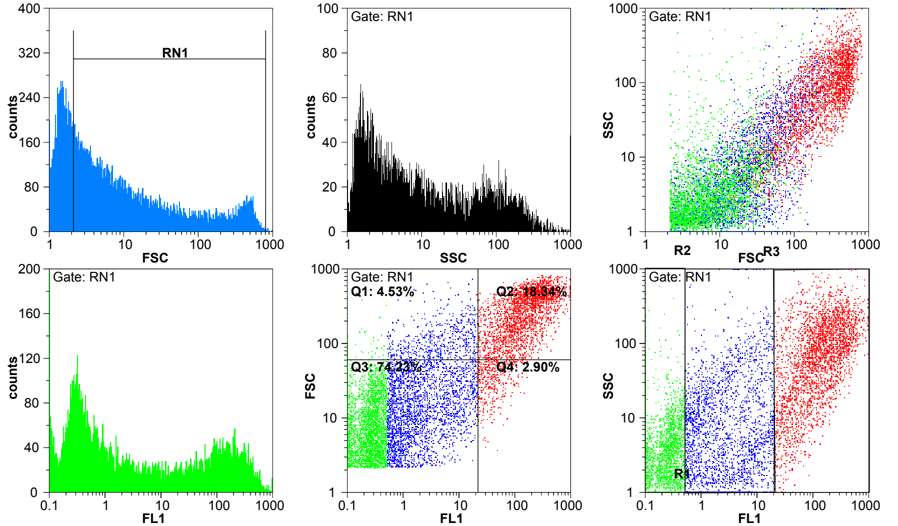

R3: 15.13%   **R1: 67.88%**   **R2: 16.98%**

Region	Gate	Count	Count/ml	%Gated	Mean-x	CV-x%	Mean-y	CV-y%
Q1	RN1	636	-	4.53	5.96	101.04	136.50	71.66
Q2	RN1	2573	-	18.34	206.46	79.57	319.24	55.12
Q3	RN1	10416	-	74.23	1.05	270.56	10.85	106.96
Q4	RN1	407	-	2.90	49.56	56.50	35.87	41.74
R1	RN1	8006	-	57.06	0.17	66.37	7.42	741.00
R2	RN1	3014	-	21.48	4.20	116.66	22.00	385.57
R3	RN1	3009	-	21.44	183.44	88.44	108.05	108.48
RN1	<None>	14032	-	70.16	73.82	193.60	-	-

timepoint 48 h plumbagin treatment

File: NNN48h_syto_004OOO.FCS   Date: 05-11-2014   Time: 09:44:28   Particles: 20000   Acq.-Time: 78 s   partec PAS

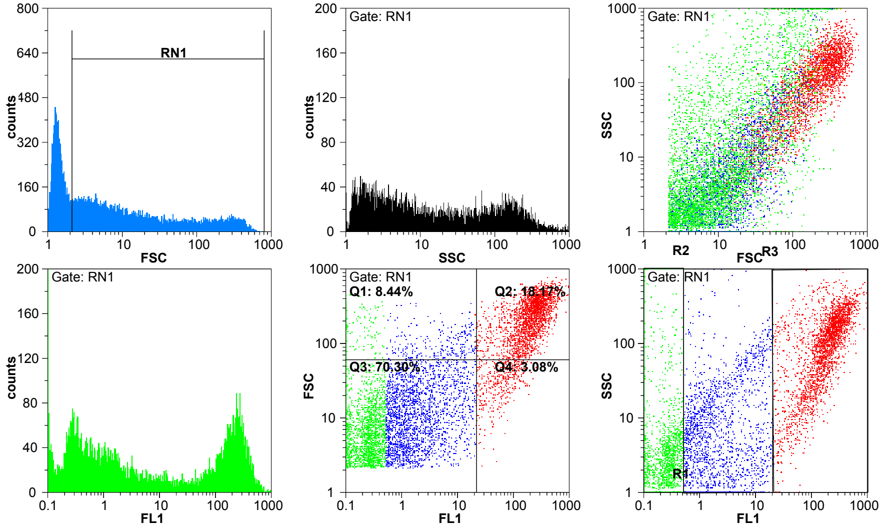

R3: 14.09%   **R1: 70.38%**   **R2: 15.51%**

Region	Gate	Count	Count/ml	%Gated	Mean-x	CV-x%	Mean-y	CV-y%
Q1	RN1	1115	-	8.44	1.78	238.65	128.60	53.24
Q2	RN1	2399	-	18.17	241.94	54.81	264.77	51.07
Q3	RN1	9283	-	70.30	0.84	273.20	11.73	103.17
Q4	RN1	407	-	3.08	75.43	60.02	34.32	43.46
R1	RN1	8070	-	61.12	0.14	65.80	48.53	329.24
R2	RN1	2317	-	17.55	3.63	119.25	19.53	389.33
R3	RN1	2815	-	21.32	217.16	63.20	138.71	84.82
RN1	<None>	13204	-	66.02	68.27	169.77	-	-
